# Structural Basis of the Membrane Association by the Conserved RocS Membrane‐Targeting Sequence in *Streptococcus*

**DOI:** 10.1002/advs.202521132

**Published:** 2026-01-15

**Authors:** Ana Álvarez‐Mena, Estelle Morvan, Clara Lambert, Anagha Kallisseri Parambil, Martin Lefeuvre, Zeren Xu, Nadia El Mammeri, Florian Malard, Erick J. Dufourc, Cécile Feuillie, Christophe Grangeasse, Birgit Habenstein

**Affiliations:** ^1^ Univ. Bordeaux CNRS Bordeaux INP CBMN UMR 5248 Pessac France; ^2^ Univ. Bordeaux CNRS Inserm IECB UAR3033, US01 Pessac France; ^3^ Université Claude Bernard Lyon 1 CNRS MMSB UMR 5086 Lyon France; ^4^ Univ. Bordeaux CNRS INSERM ARNA UMR 5320, U1212 Bordeaux France

**Keywords:** AFM, chromosome partitioning, membrane‐protein interactions, solid‐state NMR, streptococcus

## Abstract

Chromosome segregation in the human pathogen *Streptococcus pneumoniae* relies on the membrane‐binding protein RocS, which links the chromosomal DNA to the membrane. Beside the ability of RocS to bind DNA and interact with the chromosome partitioning protein ParB, little is known about how C‐terminal membrane anchor of RocS contributes to chromosome segregation. More precisely, the molecular basis of membrane targeting remains unresolved. This study addresses the interplay between RocS C‐terminal region and the lipid membrane. Combined magic‐angle spinning NMR and wide‐line NMR reveal that the membrane anchoring peptide folds into a short membrane‐inserted kink‐helix motif connected to an extended linker, with membrane insertion locally perturbing lipid packing. The fluidity of the lipid bilayer, modulated by temperature, in turn influences the anchor‐membrane interactions. At the mesoscale, AFM imaging shows that the anchor selectively associates with lipid nanodomains, clustering into discrete foci. Mutational studies further reveals that a single glycine mutation in the C‐terminal significantly perturbs chromosome segregation and alters lipid membrane properties. These findings reveal a mechanism for nanodomain association of a highly conserved membrane‐targeting motif in *Streptococci*, highlighting the kink‐helix anchor as a conserved element for membrane targeting across bacteria and beyond.

## Graphical abstract

Chromosome segregation in *Streptococcus pneumoniae* depends on RocS, a bitopic protein whose membrane‐anchoring mechanisms were unclear. Using NMR and AFM, this study reveals that the widely conserved RocS anchor binds to membranes via a conserved kink‐helix motif which inserts into lipid nanodomains. Membrane architecture and a single glycine residue critically regulate this interaction and chromosome segregation.

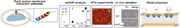

## 1 Introduction

Correct chromosome segregation is essential for the survival and proliferation of all living organisms. In most bacteria, this process is mediated by the conserved ParABS system, in which ParA and ParB cooperate with the structural maintenance of chromosomes (SMC) complex to ensure accurate DNA partitioning. We recently found that in the opportunistic human pathogen *Streptococcus pneumoniae* (the pneumococcus), which has emerged as a model organism for dissecting bacterial cell‐cycle regulation [1], chromosome segregation follows a distinct mechanism which challenges the canonical paradigm. Here, faithful chromosome partitioning during the cell cycle relies primarily on the Regulator of Chromosome Segregation (RocS) [2]. Remarkably, other molecular partners typically involved in bacterial chromosome segregation, i.e. the partitioning protein ParB and the condensin (SMC complex) remain dispensable [3], and the ParA ATPase, which generally completes the ParABS system, is entirely absent from its genome [1].

RocS mediates chromosome segregation through a dual interaction with both the chromosomal DNA and the cell membrane. It binds DNA via an N‐terminal helix‐turn‐helix (HTH) motif, oligomerizes through a central coiled‐coil domain, and anchors to the membrane over a short C‐terminal motif [2, 4]. In contrast to the well‐studied chromosome‐partitioning protein ParB [5], and although phosphorylation of the HTH domain was recently reported to regulate RocS function [2, 4], structural and mechanistic data explaining the cellular functions of RocS are still lacking. Notably, evidence shows that the ability of RocS to associate with the membrane is essential for its activity [2]. Interestingly, the short membrane anchor is reminiscent of the widely conserved cell division protein MinD membrane‐targeting sequence (MTS), which points to a potentially conserved mechanism for coordinating protein localization at the division site. Protein‐membrane interactions play a crucial role in modulating the organization and dynamics of the bacterial membranes.

Lateral segregation of membrane proteins and lipids into micro‐ or nanometer‐sized domains has first been proposed in the fluid mosaic model [6]. Since then, this model has evolved to encompass the dynamic nature of lipid and protein segregation within the membrane. In bacteria, membrane domains execute diverse functions during cell division [7], relying on the coordinated action of specific proteins and selected membrane lipids [8, 9, 10, 11]. However, studies of small peripheral membrane proteins such as RocS remain challenging due to their soft matter state upon interaction with the lipid membrane surface. To investigate the structures and interactions involved in RocS membrane association, we employed a multiscale approach, focusing on the MTS of RocS. Using a combination of magic‐angle spinning (MAS) and wide‐line solid‐sate NMR (ssNMR) together with AFM, RocS MTS insertion into the membrane bilayer was characterized in a native‐like environment. These experiments showed how the folded peptide interacts with the membrane bilayer at the atomic level, while on the mesoscopic scale, the MTS selectively associates with lipid‐enriched nanodomains. In vivo, a single mutation in *Streptococcus pneumoniae* was found to link the MTS conformation to membrane lipids and the correct chromosome segregation, supporting a mechanistic model of tightly regulated membrane anchor‐lipid interactions.

## 2 Results

### 2.1 RocS Membrane‐Targeting Sequence Folds into a Kink‐Helix Motif

**FIGURE 1:**
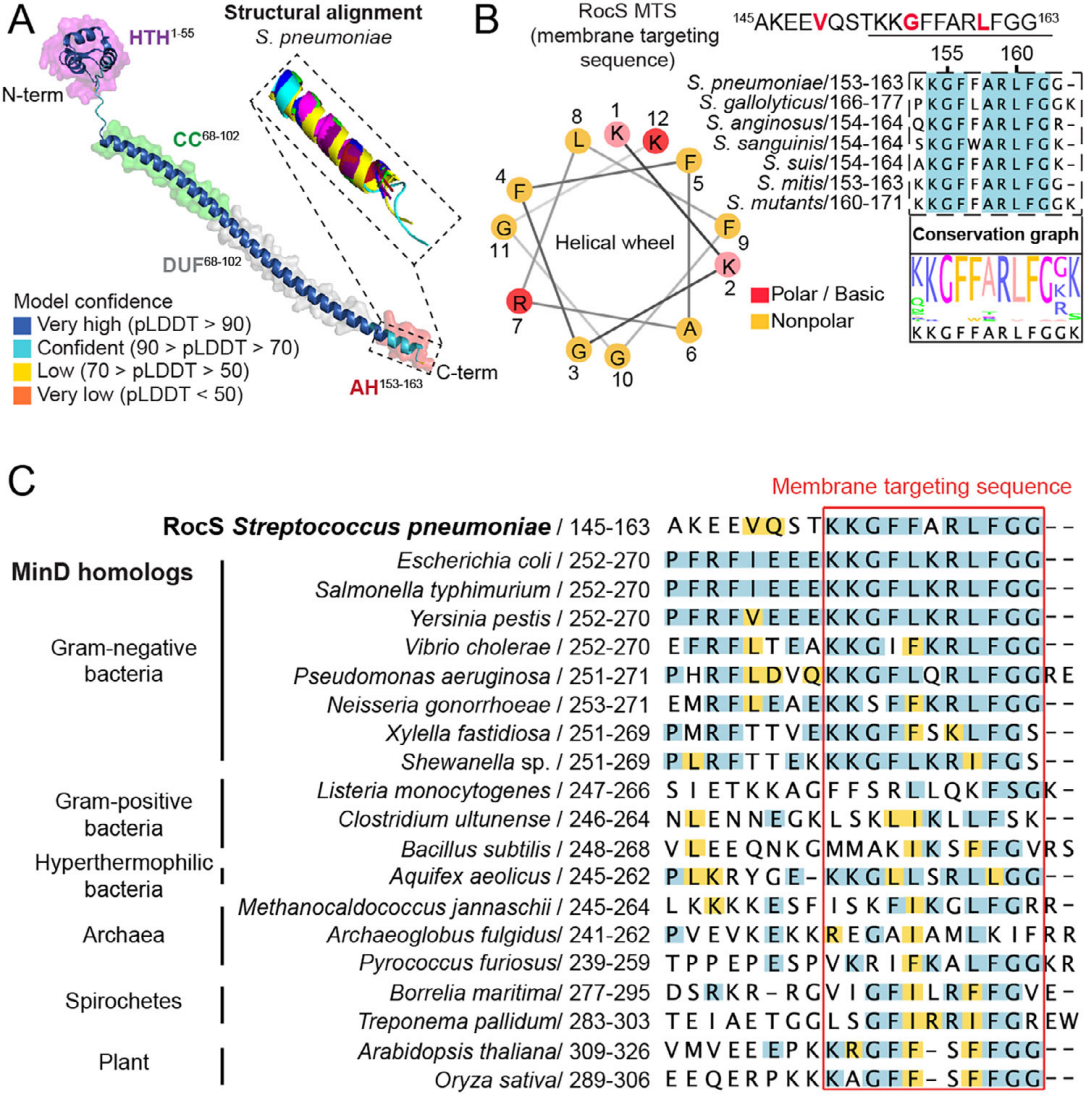
Conformational analysis of RocS and RocS MTS. A) AlphaFold‐predicted structure of RocS from *S. pneumoniae* (UniProt ID: Q8DQ15), colored according to pLDDT confidence scores. Shaded colors highlight the domain organization, including an N‐terminal HTH domain (purple), a central coiled‐coil region (green), the DUF536 (grey) and a C‐terminal amphipathic helix (red). A zoom view shows the structural alignment of the C‐terminal region across homologs from medically relevant *Streptococcus* species. The *S. pneumoniae* region used as reference is shown in blue; homologous regions are shown for *S. gallolyticus* (cyan), *S. anginosus* (purple), *S. sanguinis* (green), *S. suis* (brown), *S. mitis* (magenta), and *S. mutants* (yellow). B) Top: Primary amino acid sequence of the extended RocS membrane‐targeting sequence (RocS MTS) from *S. pneumoniae*, with residues highlighted in red corresponding to ^13^C/^15^N isotopically labeled positions selected for ssNMR studies. Middle: Multiple sequence alignment of the MTS across *Streptococcus* species. The predicted MTS is underlined in the primary sequence and fully conserved residues are shaded in light blue. Bottom: Helical wheel projection of the MTS, with polar residues highlighted in red (light red indicates residues less compatible with bilayer insertion) and non‐polar residues in yellow. C) The alignment of RocS MTS with MinD homologues shows a high conservation of the amino acid sequence. RocS (*S. pneumoniae)* is characterized by a net charge of +2 and a hydrophobic moment of 0.245 µH, whereas MinD (*E. coli*) has a net charge of +2 and a hydrophobic moment of 0.424 µH.

Membrane binding of RocS is mediated by a short MTS, which is essential for chromosome segregation in vivo [2]. To understand how this motif is integrated into the RocS sequence and to identify conserved features that may underlie its membrane‐targeting function, we performed a primary sequence alignment across *Streptococcus* species, which revealed high residue‐level conservation (Figure S1), consistent with the essential role of RocS to ensure correct DNA partitioning. Variations are mainly observed within the N‐terminal helix‐turn‐helix (HTH) motif, conferring DNA‐binding ability, as well as within the linker region connecting the HTH to the predicted coiled‐coil domain. A less conserved region links the coiled‐coil domain to the C‐terminal MTS, varying in both length and amino acid conservation. Comparison of the predicted 3D structures (AlphaFold 3, AF3) [12] reveals very similar folds, suggesting a high degree of conservation of the HTH and coiled‐coil domains (Figure 1A and Figure S2). Interestingly, the coiled‐coil domain is predicted to extend toward the C‐terminal end, with the pLDDT (predicted local distance difference test) confidence score decreasing in the C‐terminal region that encompasses the MTS. Because of its charge distribution and conservation profile, this C‐terminal segment is predicted to adopt an amphipathic helix, as illustrated by a helical wheel projection (Figure 1B), with Lysine 1 (Lys 153 of the RocS MTS) appearing less compatible with insertion into the lipid bilayer. Considering the rather perpendicular orientation of an amphipathic helix axis relative to the bilayer normal, the conserved RocS structure suggests a flat positioning of the coiled‐coil and HTH domains on the membrane surface. However, such an aligned membrane‐RocS orientation appears counterintuitive, considering that RocS is required to interact simultaneously with both the chromosome and the membrane. Interestingly, AF3 predicts a lower pLDDT confidence score within the MTS, suggesting a variability of the structure with different conformations depending on the context.

Based on the sequence conservation, we hypothesize structural plasticity in the linker region between the MTS and the coiled‐coil domain at the site of sequence divergence and the first positively charged residue of the polar‐apolar amphipathic helix, which is prone to interact with negatively charged phospholipid headgroups [13]. To further evaluate the evolutionary conservation of this motif, we performed an amino acid alignment of RocS with MinD homologs, a widely conserved cell division site‐positioning ATPase, across Gram‐negative and Gram‐positive bacteria, hyperthermophilic species, archaea, spirochetes and plants. This analysis revealed a high degree of conservation in the membrane‐targeting region, highlighting its likely fundamental role in controlling membrane localization of peripheral proteins (Figure 1C). In particular, mainly positive charge distribution and hydrophobic residue positions are well‐conserved over the distinct homologues, pointing to a dedicated role to target specific lipids or regions in the membrane.

To investigate this conserved region in detail, we focused on an extended construct, referred to as RocS MTS throughout the manuscript, encompassing the membrane‐targeting sequence underlined in the primary sequence (Figure 1B). To verify our hypothesis of structural plasticity at the linker moiety, we aimed to characterize the structural features of the membrane anchor. We first tested its solubility and recorded a solution Nuclear Magnetic Resonance (NMR) ^1^H‐^15^N correlation experiment in an aqueous environment at neutral pH 7.5. Rocs MTS reveals to be soluble and disordered, as reflected by the low signal dispersion in the proton dimension between 8 and 8.5 ppm (Figure S3). To investigate the conformational changes of RocS MTS upon insertion into the lipid membrane, we used Magic Angle Spinning (MAS) solid‐state NMR (ssNMR), which allows detection of conformations, dynamics and interactions of biomolecules in an insoluble state [14], including proteins in a near‐native membrane environment [15]. Therefore, we introduced ^13^C‐labeled residues at strategic positions within the helical wheel projection: (i) Val149, located before the polar‐apolar amphipathic helix at the N‐terminal; (ii) Gly155, the first residue likely to allow structural variations from the preceding motif (Glycine 3 in the helical wheel projection); and (ii) Leu160 (Leucine 10 in the helical wheel projection), a highly conserved residue in C‐terminal amphipathic helix (Figure 1B) exhibiting hydrophobicity comparable to that of Phenylalanine, Isoleucine and Tryptophan [16]. The residue numbers correspond to the sequence of *S. pneumoniae*.

**FIGURE 2:**
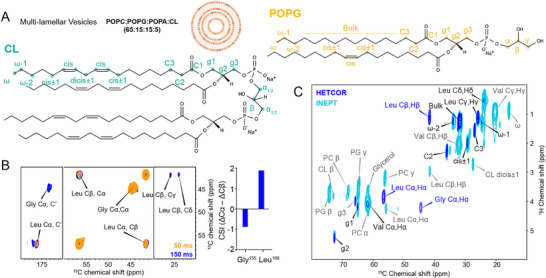
Conformational analysis of RocS MTS peptide in lipid membranes. A) Multilamellar vesicles (MLVs) were prepared using a lipid mixture of POPC, POPG, POPA, and cardiolipin (CL) at a 65: 15: 15: 5 molar ratio. The chemical structures of POPG and CL are shown, with individual atoms labeled to facilitate the interpretation of NMR chemical shifts. B) Left: Expanded views of 2D ^13^C‐^13^C PDSD ssNMR spectrum recorded at 30°C with mixing times of 50 and 150 ms, showing cross‐peaks corresponding to Gly and Leu resonances. Spectra were acquired on the isotopically labeled RocS MTS peptide reconstituted into POPC:POPG:POPA:CL MLVs at a peptide‐to‐lipid molar ratio of 1:20. Right: Chemical shift index (CSI) analysis of the observed resonances, indicating secondary structure propensities. C) Superimposed ^1^H‐^13^C HETCOR and INEPT spectra recorded at 30°C on a 600 MHz spectrometer with a MAS spinning frequency of 11 kHz. Chemical shifts observed exclusively in the INEPT spectrum are shown in grey, those unique to the HETCOR spectrum in blue, and resonances detected in both experiments in black. The chemical shift assignments are listed in Tables S1–S3.

To investigate the structure of the RocS MTS in a native‐like lipid environment, we reconstituted proteoliposomes containing *Streptococcus ssp*. phospholipids, supplemented with the zwitterionic POPC to stabilize the bilayer, and incorporated the ^13^C‐labeled peptide at a 20:1 lipid‐to‐protein molar ratio at a neutral pH 7.5 (Figure 2A; Figure S4) [17, 18, 19]. We recorded MAS ssNMR ^13^C‐^13^C correlation spectra (proton‐driven spin diffusion, PDSD) at 30 °C (as measured on the water signal [20]), relying on dipolar coupling‐based polarization transfer during the initial ^1^H‐^13^C cross‐polarization (CP) step to selectively detect immobilized peptide segments. Assignment of the Gly155 Cα and Cβ and the Leu160 Cα, Cβ and CO chemical shifts in the PDSD spectrum with a 50 ms mixing time revealed a unique, rigid conformation for these residues within the conserved C‐terminal segment, whereas no correlations were observed for the N‐terminal Val149 (Figure 2B). Increasing the PDSD mixing time to 150 ms allowed observation of signals arising from inter‐residual proximities (< approx. 4 Å) or intra‐residual side‐chain correlations. While no inter‐residual signals were detected, all correlations within the Gly155 and Leu160 spin systems were fully assigned (Figure 2B). Analysis of secondary chemical shifts (ΔδCα – ΔδCβ) of the ^13^C‐labeled residues indicates an α‐helical conformation for the C‐terminal Leu160, whereas Gly155 exhibits a kink or extended structure (Figure 2B) [21, 22].

Because in the CP‐PDSD spectra, no Val signals were visible, we hypothesized that this residue exhibits higher mobility. To test this, we recorded one‐ and 2D dipolar coupling‐based spectra (CP and HETCOR, i.e. heteronuclear correlation spectra), which selectively detect resonances of rigid structural elements. Additionally, INEPT (Insensitive Nuclei Enhanced by Polarization Transfer) [23] spectra were recorded, using scalar coupling‐based polarization transfers and thus being sensitive to mobile segments. Comparison of one‐ and 2D CP, HETCOR and INEPT spectra, recorded on complex phospholipid membranes in the absence and presence of the ^13^C‐labeled peptide, enabled unambiguous assignment of the ^1^H‐^13^C correlation signals (Figures S5 and S6) [24].

A direct comparison between the 2D HETCOR and INEPT spectra, which report on rigid and mobile residues, respectively, revealed a clear increase in mobility for Val149. All Val spin system correlations were detected in the INEPT spectrum (Figure 2C), whereas only a faint Hα‐Cα correlation was visible in the HETCOR. In contrast, Gly155 correlations were completely absent in the INEPT spectrum but detected with high intensity in the HETCOR, indicating a highly immobilized conformation. The C‐terminal Leu160 residue was observed in both spectra, with a higher relative intensity for the Hα ‐Cα correlation in the HETCOR, whereas more mobile side‐chains resonances appeared with stronger intensity in the INEPT spectra. Interestingly, the Hα chemical shift differed between the INEPT and HETCOR spectra, suggesting that a minor fraction of the INEPT signal originates from soluble species in which the chemical environment of Leu160 differs from the membrane‐embedded state. The absence of Gly correlations in the INEPT spectrum, even in the presence of a small soluble fraction, can be explained by the intrinsically reduced flexibility of the Gly residue.

To further refine the analysis of local peptide dynamics, we recorded direct pulsed ^13^C spectra (Direct Polarization, DP) with a short recycle delay of 2 sec, favorable for dynamic residues with small T1 relaxation times because the short delay precludes full relaxation of rigid residues with long T1 relaxation times during the inter‐scan delay. Comparison of the DP_2 sec_ with quasi‐quantitative DP_30 sec_ spectra with long recycle delay of 30 sec, leaving the system to fully relax, confirmed that the Val residue and the Leu side‐chains display the largest intensity differences between both spectra. In contrast, the Gly Cα signal exhibited identical intensity, underpinning the flexibility of the Val residue and the Leu side‐chain (Figure S7).

To complement the spin system assignment of Val149, relying on its higher flexibility, we recorded a PDSD preceded by a direct ^13^C pulse (DP‐PDSD), thereby omitting the CP step. As expected, in the directly pulsed ^13^C‐^13^C DP‐PDSD spectrum, the Val spin system could be partially detected and assigned (Figure S8). The ^13^C signals of the lipids were mostly visible in the INEPT spectra, consistent with the mobility of lipids acyl chains in the fluid membrane phase, whereas in the HETCOR spectra, signals corresponding to the glycerol backbone are predominantly detected, in agreement with restricted motion of the lipid headgroups within the bilayer.

In summary, our data are consistent with a kink‐helix‐like amphipathic motif centered at Gly155 of the conserved RocS MTS in a lipid membrane environment including a more mobile N‐terminal segment.

### 2.2 Interactions of the Kink‐Helix Motif with the Membrane

We then questioned how the reversible kink‐helix motif establishes contact at the interface of the phospholipid membrane. The 2D HETCOR experiment suffers from a low signal dispersion of the ^1^H dimension, precluding an unambiguous assignment of the ^1^H frequencies in a 1D proton experiment, where protein and lipid acyl chain resonances superimpose. MAS ssNMR methods, however, allow selective detection of water‐protein and membrane‐protein interactions [25, 26].

To detect correlations between mobile protons, including water and the hydrocarbons of the acyl chain, and the inserted kink‐helix motif, we employed an extended 2D ^1^H‐^13^C HETCOR experiment. Scalar dephasing during the T_2_ filter suppresses polarization on directly ^13^C‐bound ^1^H, mainly from the peptide [25, 27, 28], causing relaxation of rigid protons while preserving signals from mobile protons, including water and lipid CH_2_ groups. Following t1 chemical shift evolution, a 100 ms mixing period allows equilibrate magnetization through ^1^H spin diffusion prior to the ^1^H‐^13^C CP transfer [29, 30, 31].

**FIGURE 3:**
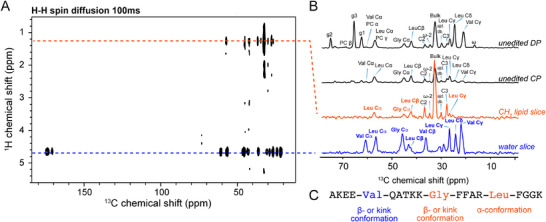
2D ^1^H‐^13^C HETCOR spectrum indicate that the RocS MTS C‐terminal helix inserts into the surface or the hydrophobic core of lipid membranes. A) 2D ^1^H‐^13^C HETCOR spectrum of the RocS MTS peptide reconstituted into POPC:POPG:POPA:CL MLVs [31], recorded at 31°C on a 600 MHz spectrometer with a MAS frequency of 11 kHz. B) ^13^C cross sections at the lipid CH_2_ and water ^1^H chemical shifts are shown. Unedited DP and CP are shown for comparison. Key protein and lipid ^13^C signals are assigned. Lipid acyl chain ^1^H to Leu and Gly ^13^C correlations are observed, indicating that the RocS MTS inserts into the hydrophobic interior of the membrane near the terminal carbon atoms of the hydrocarbon chain. The spectrum was measured using a ^1^H mixing time of 100 ms after a ^1^H T2 filter of 2 x 2.18 ms to remove the rigid proton signals (i.e., mostly protein protons). C) Amino acid sequence of the RocS MTS peptide. The three ^13^C‐labeled positions are colored based on their membrane insertion depth and their chemical shift derived conformations are specified (see Figure 1B). The C‐terminal Leu residue resonances reflect the helical conformation within the lipid bilayer.

As expected, this extended ^1^H‐^13^C HETCOR spectrum shows intense signals at the ^1^H resonance frequencies of mobile water and lipid CH_2_ groups (Figure 3A). On the CH_2_ frequency, correlations from Gly Cα, Leu Cα and Leu Cγ are observed, demonstrating clear insertion of the kink‐helix motif in the surface or into the hydrophobic core of the bilayer. Val resonances, in contrast, are absent at the CH_2_ frequency, indicating that Val149 is distant from the hydrophobic membrane core (Figure 3B). Notably, Val Cα and Cγ lead to an intense signal at the water frequency, underpinning that the N‐terminal Val is exposed to water while absent from the membrane core (Figure 3B). Secondary chemical shift analysis of Val149 suggests an extended or kink conformation (Figure 3C), while its high intensity in the INEPT spectra (Figure 2C) highlights the residual flexibility of this conformational segment.

To support membrane insertion of the MTS and its structural plasticity, we performed all‐atom molecular dynamics simulations (MD). Starting from the AF3 predicted helical peptide structure (Figure 1A), positioned at ≈10Å distance from the surface of a membrane bilayer, we monitored the progressive insertion of the MTS into one leaflet of the membrane, where it established contacts with the hydrophobic acyl chains (Figure S9A). For all three replicate simulations, the peptide became associated to the membrane within a few hundreds of nanoseconds, then it remained stably associated for the remainder of the 5 µs simulations. Importantly, all simulations exhibited recurrent, transient helix bending around a kink at residue Gly155 (Figure S9B,C). In this configuration Leu160 remains in contact with the hydrophobic region of the membrane leaflet, while the N‐terminal Val149 partially extends toward solvent‐accessible regions. The MD results support the experimentally observed conformational preferences and the contacts of the MTS with the membrane bilayer.

**FIGURE 4:**
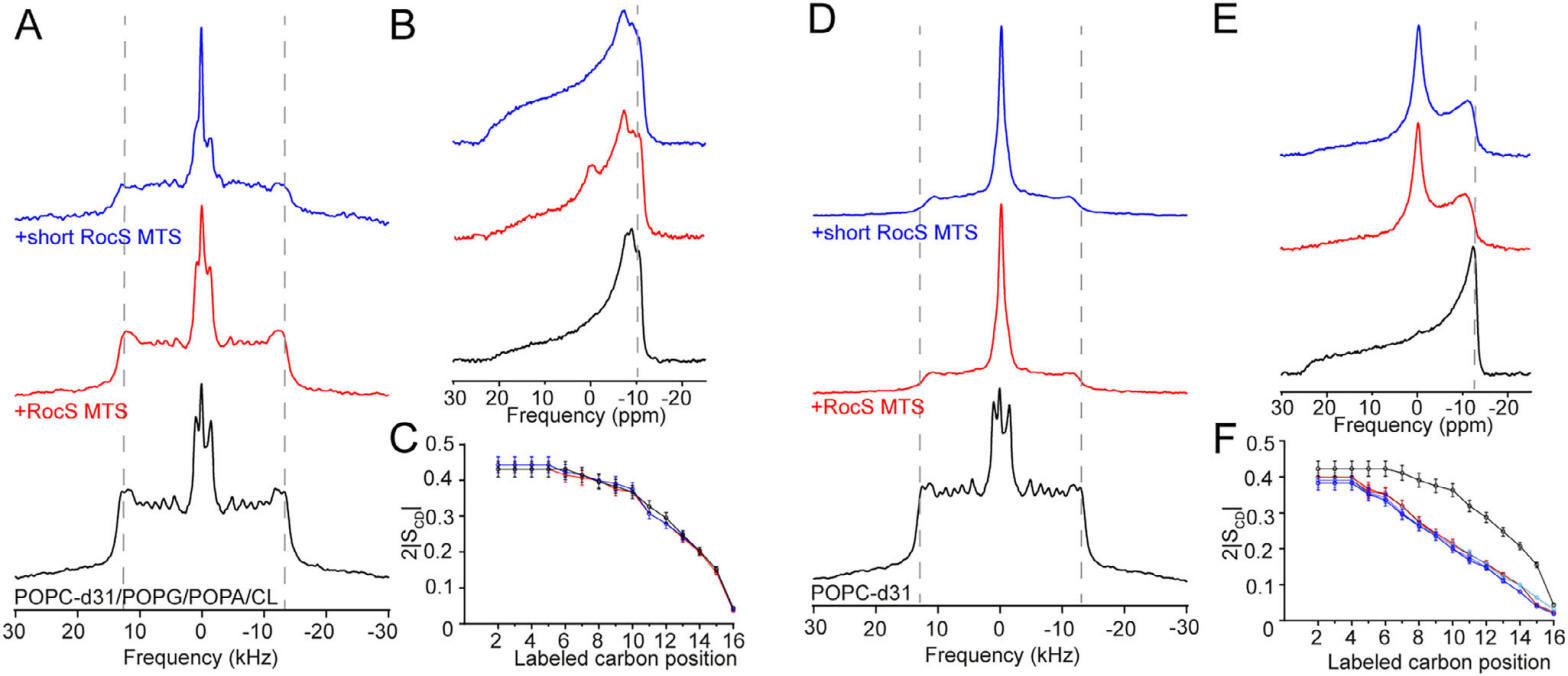
Membrane interactions of RocS MTS and short RocS MTS by wide‐line ssNMR. A) ^2^H spectrum detecting the quadrupolar splitting on the symmetric doublets, recorded on POPC:POPG:POPA:CL vesicles doped with POPC‐d31 at 298K (blue: short RocS MTS, red: RocS MTS, black: control). Dashed lines serve as eye guidance. B) ^31^P spectrum showing the chemical shift anisotropy of the phosphorus lipid head groups (same color code as in A). Dashed lines serve as eye guidance. C) The carbon‐deuterium order parameters 2*|S_CD_| of ^2^H_31_‐POPC in the POPC:POPG:POPA:CL vesicles. Error bars indicate a ±5% uncertainty, reflecting methodological and simulation‐related variability [19]. D–F) are recorded on RocS MTS in contact with POPC (doped with POPC‐d31) membranes and represent the data equivalent to A‐C. Color codes are equivalent for A and B. In (F), three distinct simulations of the order parameters 2*|S_CD_| have been performed per RocS MTS variant (Figure S10C) because of the lower spectral quality of the ^2^H spectrum of POPC vesicles resulting from a high fraction of small objects, reflected in the intense isotropic line (shades of blue: short RocS MTS, shades of red: RocS MTS).

To investigate the impact of RocS MTS on phospholipid membranes, we recorded wide‐line ssNMR on ^2^H and ^31^P labeled nuclei. ^2^H‐detected ssNMR spectra reveal the quadrupolar splitting of the deuterium, so‐called Pake doublet, along the deuterated acyl chain (POPC‐^2^H_31_ was used), which depends on membrane phase and local dynamics and can be translated into the C‐^2^H order parameter. The axially symmetric ^2^H spectrum reflects the fluid lamellar membrane phase (ld) of the vesicles, preserved in the presence of RocS MTS (Figure 4A). The outer quadrupolar splittings Δν_Q_ correspond to the first seven C‐^2^H positions along the POPC‐^2^H_31_ acyl chain, whereas narrower splittings and the flexible methyl group are detected in the smallest doublet [32, 33, 34].

Two peptides were tested: a truncated variant lacking the C‐terminal Lys (AKEEVQATKKGFFARLFG), referred to as short RocS MTS, and the full‐length peptide containing the terminal Lys (AKEEVQATKKGFFARLFGGK), referred to as RocS MTS, which increases the amphipathic character of the peptide (Figure 1B). Larger quadrupolar splittings reflect reduced mobility of the acyl chain near the sterically constrained glycerol backbone. Both spectra exhibit similar widths and peak positions, indicating that the RocS MTS and short RocS MTS do not modify the acyl chain mobility during membrane insertion (Figure 4A). The ^31^P chemical shift anisotropy (CSA) confirms the lamellar phase of the lipid vesicles both in the absence and presence of RocS MTS variants (Figure 4B) [35]. Minor shifts of the ^31^P signals suggest that both RocS MTS variants interact with lipid headgroups, modifying their chemical environment, consistent with direct interactions of the positively charged Lys residues with the anionic lipid headgroups (Figure 4B). Interestingly, the ^31^P line shape of the complex membrane in the presence of short RocS MTS shows reduced deformation under the external magnetic field compared to the complex membrane alone, consistent with reinforcement of the membrane shape by the short RocS MTS (Figure S10B) [36]. In contrast, RocS MTS induces a small fraction (∼3%, from spectral simulations) of reduced‐size objects [35], i.e. smaller membrane vesicles undergoing isotropic tumbling, as detected in the isotropic central ^31^P signal (Figure S10B). This effect is observed only for RocS MTS and not for short RocS MTS, likely reflecting stronger interactions due to the additional terminal Lys. The local order parameter|S_CD_| along the acyl chain can be derived from the quadrupolar splittings Δν_Q_ (Figure S10A) [17, 19]. Comparison of the acyl‐chain order parameters in the absence or presence of RocS MTS reveals a similar acyl chain mobility, indicating that the fluid lamellar phase is preserved upon RocS MTS insertion (Figure 4C).

Because we could not exclude the possibility of lipid segregation in the lipid vesicles using POPC‐^2^H_31_ as a probe, we then tested the effect of both RocS MTS variants on vesicles composed only of POPC, doped with POPC‐^2^H_31._ Both RocS MTS and short RocS MTS significantly reduced the width of the POPC ^2^H doublets, indicating a destabilizing effect of the peptides on the acyl chains of POPC membranes (Figure 4D). Furthermore, the pronounced isotropic central ^31^P signal (Figure 4E) indicates the presence of a substantial fraction of smaller, fast tumbling objects, such as micelles (36% and 34% for RocS MTS and short RocS MTS, respectively, as derived from spectral simulations (Figure S10D). Consistent with this destabilizing effect, the acyl chain order parameters |S_CD_| are significantly reduced, with both RocS MTS variants affecting |S_CD_| up to the central carbons of the membrane (Figure 4F). Such disruptive effects of amphipathic helices and their dependence on phospholipid composition have been reported previously for antimicrobial peptides [37, 38, 39]. Taken together, these results indicate that, compared to the effects on POPC membranes, the complex phospholipid composition stabilizes the membrane upon insertion of RocS MTS or short RocS MTS.

### 2.3 Temperature‐Controlled Membrane Targeting of the Kink‐Helix Motif

MAS ssNMR experiments performed at lower temperatures are commonly used to enhance sensitivity by taking the advantage of multiple parameters, such as changes in the spin state distribution, reduced molecular dynamics, longer longitudinal relaxation times and more efficient dipole‐based polarization transfers [40]. To optimize polarization transfer within membrane‐bound RocS MTS, the sample temperature was decreased at a spin rate of 11 kHz from 292 K, corresponding to a sample temperature of ∼31 °C, estimated from the chemical shift of the water signal [20] to ‐1 °C, with an intermediate point at 12 °C.

**FIGURE 5:**
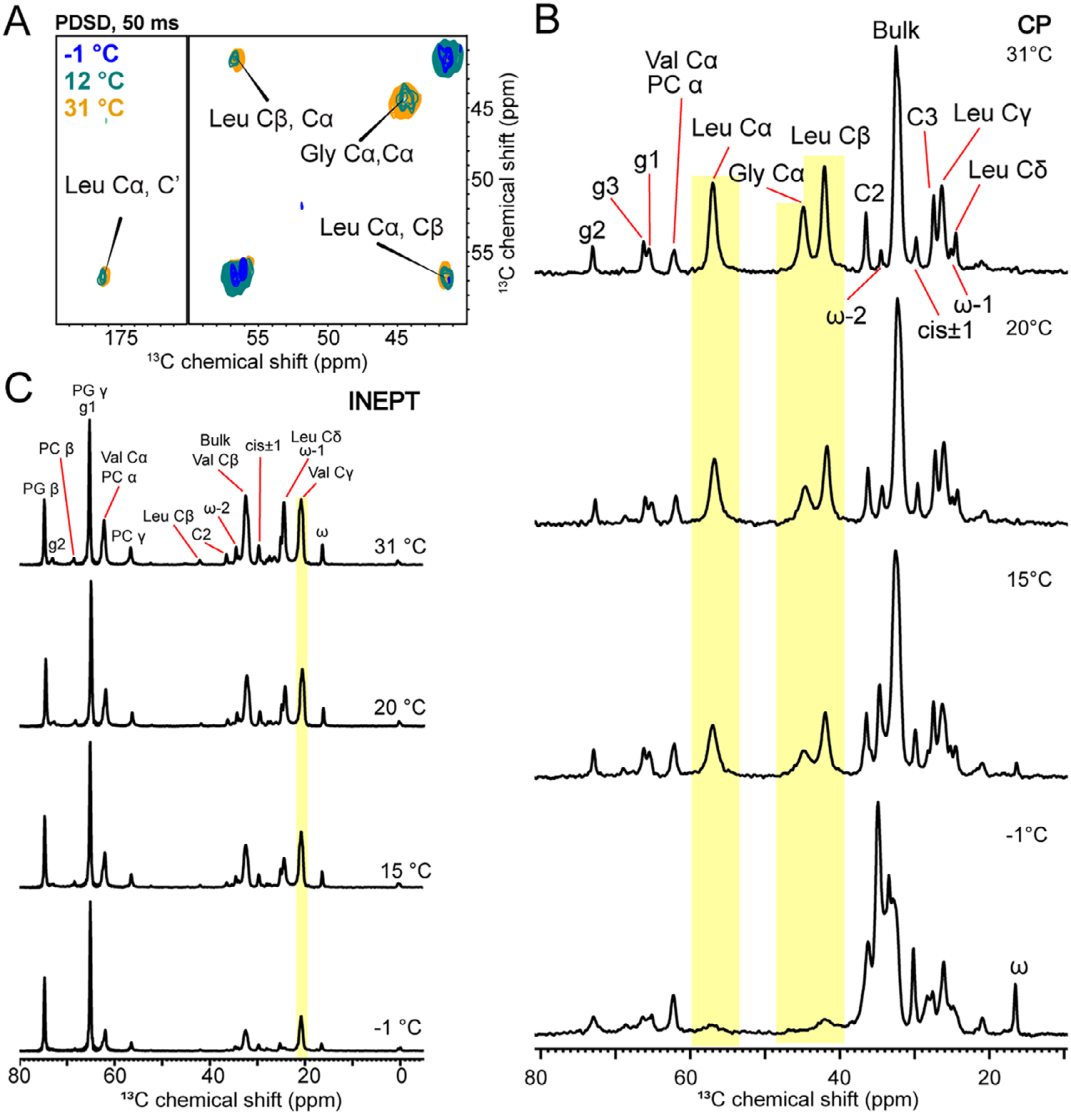
Temperature dependency of RocS MTS membrane binding. A) Superimposed 2D ^13^C‐^13^C PDSD spectra of the isotopically labeled RocS MTS peptide reconstituted into POPC:POPG:POPA:CL MLVs at a peptide‐to‐lipid molar ratio 1:20, recorded with a 50 ms mixing time at ‐1 °C, 12 °C, and 30 °C. Cross‐peaks corresponding to Leu and Gly residues are highlighted. B) 1D ^13^C cross‐polarization (CP) spectra of the same sample, acquired at 31 °C, 20 °C, 15 °C, and ‐1 °C. Chemical shifts corresponding to lipid and labeled amino acid ^13^C resonances. CP highlights the rigid components of the sample. C) 1D ^13^C INEPT spectra recorded under identical conditions as in B. INEPT selectively detects the mobile regions of the peptide and the membrane. All spectra were acquired on a 600 MHz spectrometer with a MAS spinning frequency of 11 kHz.

Unexpectedly, we observed a progressive loss of signal intensity in the 2D ^13^C‐^13^C CP‐PDSD spectra upon cooling (Figure 5A). At 12 °C, correlations corresponding to Gly and Leu spin systems were still detectable, albeit with lower intensity, while at ‐1 °C only a weak off‐diagonal signal on the noise level, potentially corresponding to the Leu side‐chain Cα‐Cβ transfer, remained. The linewidths of the signals detected in the 2D PDSD are reflective of T2 relaxation and are directly affected by the structural homogeneity of the rigid elements in the membrane‐bound peptide. The linewidths in the 1D traces of the 2D PDSD at 57 ppm, centered on the Leu Cα position, increase from 113 Hz to 140 Hz for the Cα‐Cβ correlation, when decreasing from 31 °C to 12 °C and no signal is detected at ‐1 °C (Figure S11). While the linewidth thus increases only moderately, the signal intensity decreases rapidly below the limit of detection at ‐1°C in the 2D PDSD. The increasing linewidth could stem from previously invisible conformational variability, reduced in dynamics and thus contributing to the chemical shift distribution, while the clear loss of signal indicates more dynamics in the peptide at the position of the Leu. The signal loss and moderate increase in linewidth is equally observed for the Gly Cα position (134 Hz, 31 °C vs. approx. 250 Hz, 12 °C), however, precise measurements at 12 °C were difficult due to the low signal intensity.

To confirm that the observed effects were not due to spin diffusion, we took advantage of the resolved peptide signals in the CP spectrum (Figure S5) and recorded 1D CP spectra. The Leu Cα, Leu Cβ and Gly Cα signals progressively decreased with decreasing temperature (Figure 5B). In contrast, the resonances of the lipid acyl chains shifted but retained similar intensity, as expected for temperature‐induced chemical shielding changes [41]. The pronounced reduction in peptide signal intensity therefore indicates a loss of conformational confinement of the RocS MTS, consistent with a loss of membrane anchoring as the lipid bilayer approaches the fluid‐to‐gel phase transition, evidenced by the resonance shifts of the acyl chains resulting from altered chemical shielding in the gel phase [41]. However, based on our data, we cannot clearly differentiate between differences in dynamics and relaxation of a potentially still membrane‐bound peptide or the dissociation of the peptide from the membrane. Importantly, this effect was fully reversible, with complete recovery of the initial signal intensity upon reheating to 31 °C.

The ^1^H ‐^13^C INEPT spectra reported clearly only the Val Cγ resonance, as the other ^13^C signals could not be unambiguously assigned to peptide resonances (Figure S6). In agreement with the general decrease in signal intensity in the INEPT spectrum due to reduced molecular motion, the Val Cγ resonance follows the same trend, indicating that its motional state is affected by the temperature and decreases on a comparable scale (Figure 5C).

### 2.4 Nanodomain Clustering of the Kink‐Helix Motif

**FIGURE 6:**
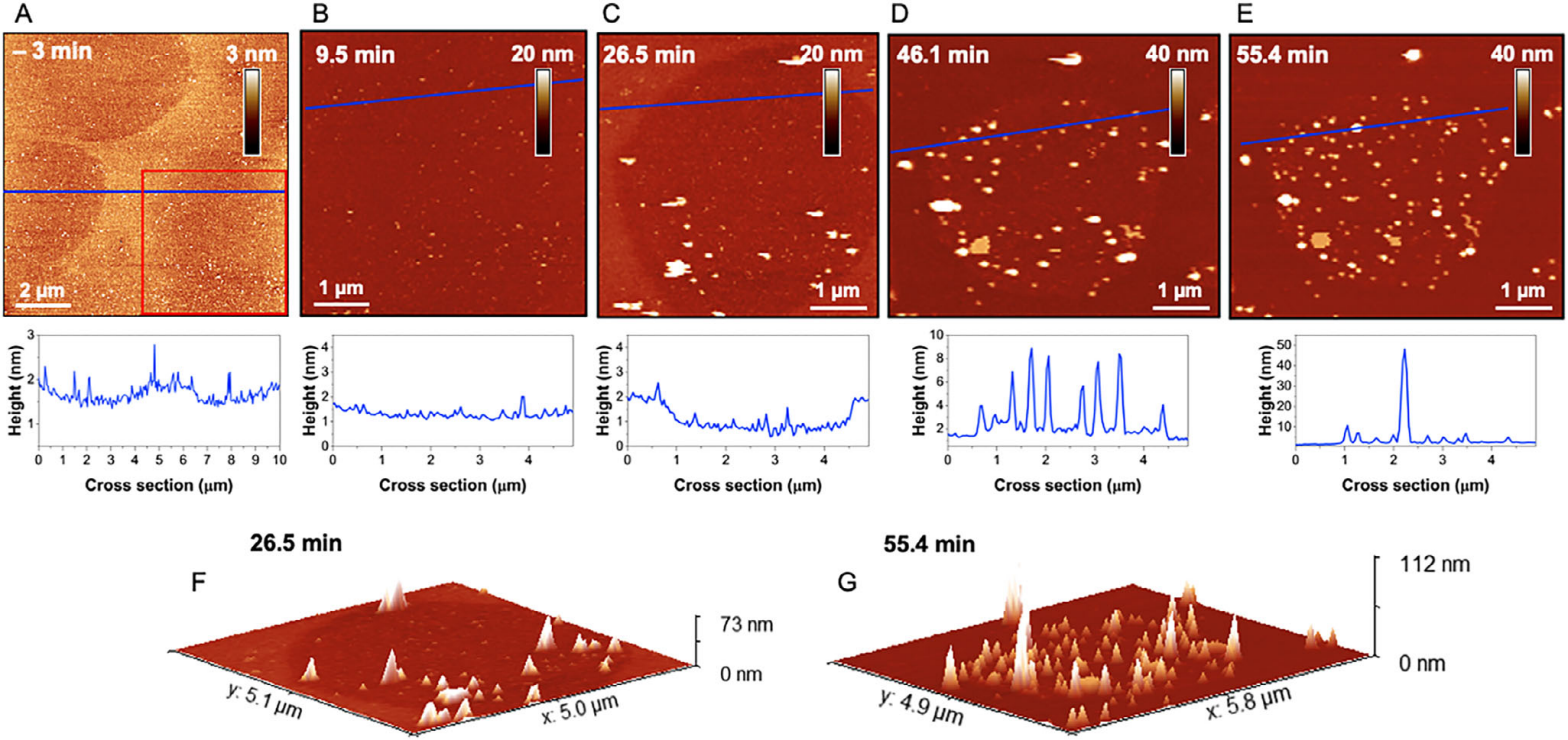
AFM imaging of the short RocS MTS ‐ membrane interaction (10 µM of short RocS MTS, lacking the C‐terminal Lys residue). Selected images of the sequence are presented chronologically, before (A, 3 min before) and after addition of 10 µM of short RocS MTS (respectively 9.5, 26.5, 46.1, and 55.4 min after addition of the peptide for panels B to E). For each panel, a cross section is presented, corresponding to the blue line, as well as 3D views of panels c and e for time points t+26.5 min (F) and t+55.4 min (G).

We next investigated the association of RocS MTS with the membrane at the mesoscopic scale. Previous studies, including our own, have shown that AFM on supported lipid bilayers (SLBs) allows detailed examination of membrane topology, including the formation of nano‐ and micrometer‐sized lipid domains and protein‐membrane interactions [42, 43, 44, 45]. SLBs were prepared with the same lipid composition as used in MAS ssNMR experiments, and AFM imaging confirmed their stability for over 30 min prior to RocS MTS addition (Figure S12). We observed a stable distribution of slightly thinner nanodomains across the SLBs, with a height difference of 0.6 +/‐ 0.2 nm relative to the surrounding bilayer. These domains initially exhibited diameters of 1.35 +/‐ 0.43 µm (Figure 6A; Figure S13) and remodeled over time (Figure S14).

Both peptide variants were tested: the short RocS MTS, lacking the C‐terminal positively charged Lys residue, and RocS MTS, the full peptide (Figure 1B). RocS MTS preferentially associated with the preformed nanodomains for both variants. Tracking a ≈5 x 5 µm^2^ membrane area (red square) after addition of 10 µM short RocS MTS, we observed membrane remodeling: the lower domains became more prominent after 26 min of incubation, with a height difference of 1.3 nm. Simultaneously, protein accumulation occurred specifically on the lower domains (Figure 6B,C,F). After 46 min, the initially lower domains protruded slightly, by ∼0.5 nm relative to the surrounding membrane (Figure 6D,E). This behavior was consistently observed, with protein accumulation on the lower membrane domains and domain protrusion as early as 17.5 min after addition of 10 µM short RocS MTS (Figure S15). Distinct clusters were repeatedly observed, reaching heights of 10‐20 nm after 46 min of incubation, with average diameters of 112 nm +/‐ 24 nm. After 55 min, some clusters reach 50 – 60 nm in height (Figure 6E,G).

The full RocS MTS, containing the terminal Lys, showed similar preferential association with thinner nanodomains. Notably, incubation of the RocS MTS peptide at 10 µM or 5 µM led to rapid membrane coverage, with protein accumulation observed after only 18 min at 10 µM and 29 min at 5 µM (Figure S16), indicating a stronger propensity to interact with the lipid bilayer compared to the short RocS MTS. This behavior is consistent with the presence of an additional positively charged residue, which reinforces the amphipathic character and the tendency to interact with anionic lipids. At 0.5 µM, the peptide selectively accumulated on the thinner bilayer domains, forming clusters with individual heights of 15 – 30 nm after 88 min of incubation (Figure S17). After 88 min, these clusters exhibited an average diameter of 79 nm +/‐ 11 nm, approximately 30% smaller than those observed for the short RocS MTS. Collectively, these observations indicate that RocS MTS organizes into multimeric clusters on the lipid membrane, consistent with our previous findings that RocS forms dimers or higher order multimers to effectively promote chromosome segregation [2].

### 2.5 Glycine 155 from the Membrane‐Targeting Motif is Critical for Chromosome Segregation

**FIGURE 7:**
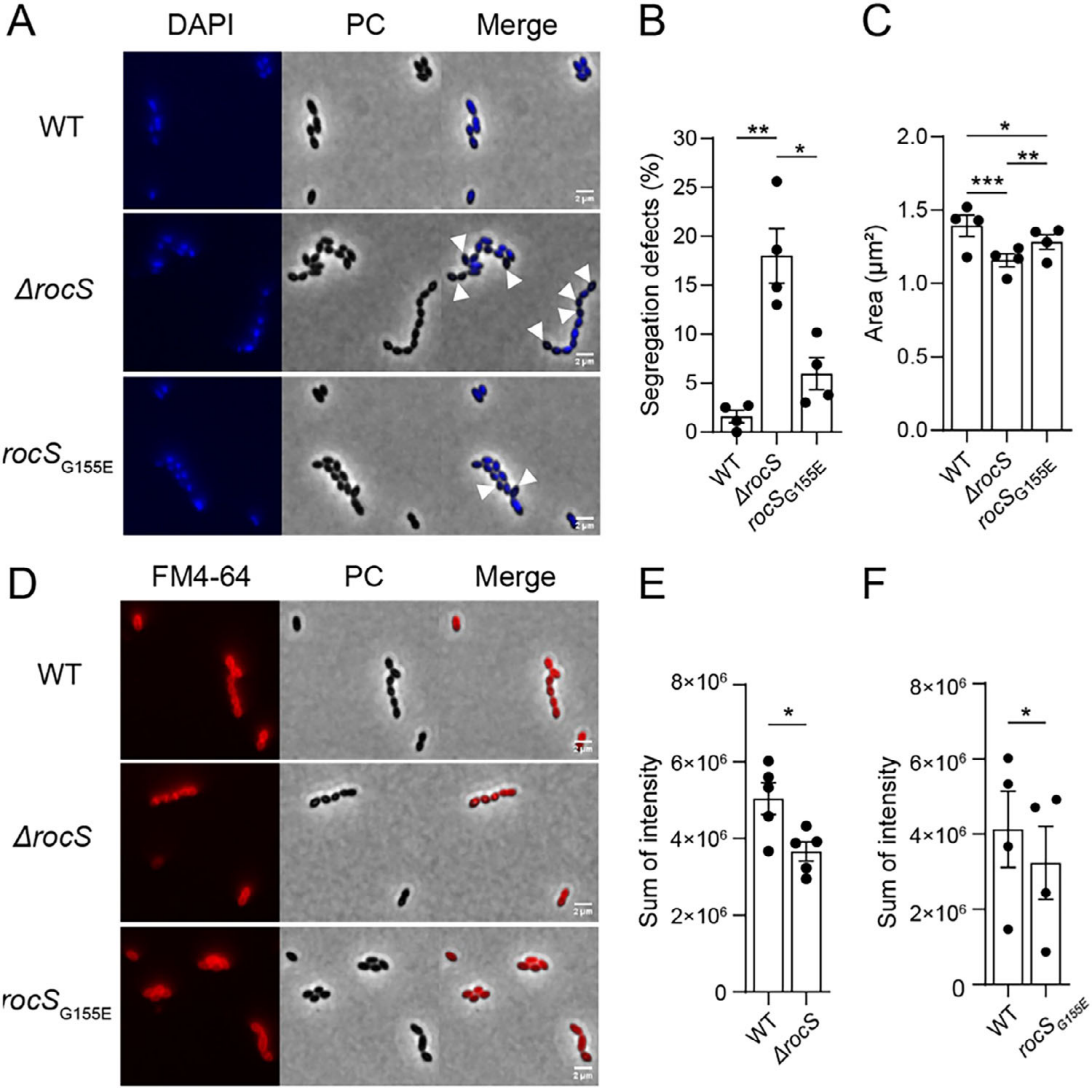
Chromosome segregation defects and altered cell morphology in *rocS*_G155E_. A) Detection of DNA (stained with DAPI) and phase contrast images (PC) of wild‐type, *ΔrocS* and *rocS_G155E_* pneumococcal cells. The arrowheads indicate anucleate cells. Images are representative of four experiments repeated independently. Scale bar, 2 µm. B) Bar chart representing the percentage of chromosome segregation defects observed in the wild‐type, *ΔrocS* and *rocS_G155E_* strains. Bars represent the mean (± SEM) of four independent experiments. The strains were compared using a one‐way ANOVA test with a Bonferroni post‐test (p‐value: **, 0.0025; *, 0.0120). C) Bar chart representing the cell areas of wild‐type, *ΔrocS* and *rocS_G155E_* cells. Bars represent the mean (± SEM) of four independent experiments. The strains were compared using a one‐way ANOVA test with a Bonferroni post‐test (p‐value: ***, 0.0003; **, 0.0078; *, 0.0144). D) Membrane labelling with FM4‐64 and phase contrast images (PC) of wild‐type, *ΔrocS* and *rocS_G155_*_E_ pneumococcal cells. Images are representative of five for *ΔrocS* and four for *rocS*_G155E_ experiments repeated independently. Scale bar, 2 µm. E‐F) Bar chart representing the sum of FM4‐64 intensity in the *S. pneumoniae* wild‐type, *ΔrocS* (E) and *rocS_G155E_* strains (F). Bars represent the mean (± SEM) of respectively five (E) and four independent experiments (F). The strains were compared using a paired t‐test (p‐value: *, 0.0529 for G and *, 0.0283 for H).

Given the pronounced membrane interactions of the kink‐helix motif in vitro, we next sought to validate its functional role in vivo. Specifically, we asked whether perturbing Gly155, the key residue forming the kink in the RocS MTS would affect chromosome segregation in *S. pneumoniae*. To address this, we analyzed a mutant strain carrying the G155E point mutation using DAPI staining (Figure 7A), with wild‐type and *∆rocS* strains as controls. As shown in Figure S18A, expression of *rocS‐G155E* fused to the *gfp* was not significantly altered compared to *rocS‐gfp*. As expected, 99% of WT cells displayed efficient chromosome segregation, whereas 20% of *∆rocS* cells were anucleate, reflecting defective segregation (1, 3). Remarkably, mutating Gly155 to the negatively charged Glu also caused defective segregation, with 7% of anucleate cells (Figure 7B). Consistently, the cell area was reduced in both *∆rocS* and *rocS*_G155E_ strains (Figure 7C), indicating that Gly155 affects RocS function during chromosome segregation, underpinning its major role during membrane targeting. Supporting this, the cellular localization of RocSG155E‐GFP was strongly altered compared to of RocS‐GFP (Figure S18B,C). This phenotype might result from perturbation of the kink‐helix conformation, the introduced negative charge at the kink position or a combined effect of both.

Given the strong effect of this single residue, we hypothesized that *ΔrocS* and the G155E mutation might also impact membrane lipid homeostasis. To test this, we monitored FM4‐64 incorporation (Figure 7D), an amphipathic dye targeting the hydrophobic core of the membrane [46]. FM4‐64 fluorescence was reduced in *ΔrocS* cells relative to WT cells (Figure 7E), indicating altered membrane lipid content and suggesting decreased levels of negatively charged phospholipids that facilitate FM4‐64 incorporation [47]. A similar reduction was observed in *rocS*_G155E_ cells (Figure 7F), demonstrating that perturbation of Gly155 similarly modulates membrane properties. However, the current data cannot distinguish changes in membrane composition from changes in the membrane architecture alone.

Overall, these findings establish Gly155 as essential for both chromosome segregation and proper membrane organization, corroborating the membrane‐targeting function of the kink‐helix motif observed in vitro.

## 3 Discussion

Predicted structures of the RocS protein family indicate a conserved elongated helix, despite the structural plasticity required for coordinating membrane binding and chromosome segregation during the *Streptococcus* cell cycle. It was previously proposed that RocS anchors at the membrane near the division site, where it interacts simultaneously with the chromosome and the membrane. As cell division progresses, RocS migrates along with the OriC region, maintaining these dual interactions with the nucleoid and the membrane. (Figure 4A in ref. [2]). Previous observations also demonstrated that dimer or higher‐order multimer formation is essential for RocS function [2, 4]. We therefore hypothesized that a specific structural element, distinct from a continuous helix, connects the membrane‐targeting sequence to the oligomerizing coiled‐coil domain.

To verify this hypothesis, we used MAS ssNMR and analyzed RocS MTS reconstituted into phospholipid vesicles and revealed that the highly conserved Gly155, proposed to be part of the amphipathic helix and located adjacent to two Lys residues at the N‐terminus of RocS MTS [2], can adopt a kink conformation upon membrane association. This kink is immediately followed by the C‐terminal amphipathic motif, as evidenced by the well‐defined helical conformation of the hydrophobic Leu160 residue. The kink‐helix motif forms only when RocS MTS interacts with the membrane surface, remaining disordered in solution, as previously reported for isolated amphipathic helices [13]. In contrast to the helical motif, the N‐terminal region of the RocS MTS, represented by Val149, remains flexible on the microsecond timescale compared with the rigid kink‐helix motif.

Taking advantage of MAS ssNMR to probe direct acyl chain‐peptide interactions [27, 30, 31], we show that the kink‐helix motif is, at least partially, in contact with the hydrophobic membrane as evidenced by equilibrated H‐H spin diffusion. Combined with the analysis of RocS MTS dynamics from directly pulsed ^13^C‐detected spectra, our data support a model in which the Leu side chain may extend toward the hydrophobic membrane region, adopting a flexibility comparable to that of the acyl chains.

Having established the conformation of the RocS MTS on the membrane, we next examined how changes in the membrane physical state influence its conformation and dynamics. Lowering the temperature effectively modified the membrane lipid environment near the fluid‐to‐gel phase transition, as monitored by MAS ssNMR on the lipid CH_2_ signals, without freezing the molecular motion of the flexible N‐terminal RocS MTS. Interestingly, the temperature gradient led to a marked decrease in MAS ssNMR signal intensity for the RocS MTS, indicating that the membrane‐bound peptide may partially dissociate from the membrane, thereby losing its kink‐helix conformation. We can, however, not exclude that the peptide remains associated to the membrane, exhibiting different dynamics. Because this loss of structure was reversible upon restoring the temperature to its initial value, we infer that the fluid phase of the membrane plays a key role in promoting MTS‐membrane interactions.

Wide‐line NMR further confirmed that the kink‐helix motif interacts with the lipid bilayer without perturbing the lamellar phase of complex phospholipid membranes. These findings substantiate a model in which the RocS MTS localizes at the membrane surface, with the Leu side chain inserting into the hydrophobic region of the bilayer while preserving the overall order and stability of the membrane. In contrast, in simple POPC vesicles, RocS MTS destabilized the membrane, leading to the formation of small objects, reflecting the common disruptive potential of the amphipathic helices [37, 38, 39]. Our data thus reveal that the complex phospholipid composition of bacterial membranes stabilizes RocS MTS insertion, preventing large scale membrane disordering effect and preserving bilayer integrity. However, the impact of RocS on membrane fluidity may differ in presence of its additional structural domains, namely the coiled‐coil and HTH domains [19].

To examine how the structural organization of the RocS MTS at the membrane surface translates to larger‐scale membrane interactions, we performed real‐time AFM imaging, which revealed the stable formation of thinner lipid nanodomains in the POPC:POPG:POPA:CL membrane bilayer, suggesting increased fluidity. The RocS MTS kink‐helix motif efficiently associates with these preformed nanodomains, forming 80 nm wide clusters that grow to >100 nm for the short RocS MTS, maintaining a constant size after approximately 1 hour, reminiscent of the foci observed in vivo [2]. Notably, the partially positive charged C‐terminal residue, which reinforces the amphipathic character of the helix, significantly enhances the propensity of the kink‐helix motif to interact with the SLB. Amphipathic helices are widely distributed in cells to target peripheral membrane proteins to specific membranes, often serving as curvature sensors, as exemplified by the well‐studied long α‐synuclein [48]. In contrast to α‐synuclein, the RocS kink‐helix motif is short (≈10 residues) but similarly carries a positive net charge (net charge of 2 at neutral pH). Consistent with general principles, proteins containing amphipathic helices preferentially bind to regions of increased fluidity (RIFs) in complex membranes due to easier insertion into the lipid bilayer [7]. Our results demonstrate that the RocS kink‐helix MTS preferentially associates with thinner lipid nanodomains. Given the structural features of the here‐used CL, including lipid shape, headgroup size and two unsaturations per acyl chain, we can expect CL to be lower in height compared to POPC and POPG, which have the same acyl chains as POPA but bigger headgroups. Lower domains enriched in CL would therefore be consistent with both its size and shape, and its low Tm (< ‐20 °C) [49]. Because cardiolipin (CL) furthermore participates in bacterial cell‐division site selection [50] and forms RIFs in vivo and in vitro [51], we propose that the RocS kink‐helix MTS could mediate association with fluid nanodomains, enriched in CL. Considering the relatively low CL content in the membranes studied here (5%), we further speculate that these nanodomains may generally contain a higher proportion of anionic lipids.

Importantly, in vivo validation mirrors these in vitro findings. Perturbing the structural plasticity mediated by the conserved Gly155 residue within the MTS leads to chromosome segregation defects even more severe than those caused by deletion of the essential partner ParB [2], underscoring the central role of the Gly155 kink in RocS function. This mutation establishes a direct link between RocS, the kink motif and the membrane lipid composition of *S. pneumoniae*. Interfering with RocS function alters the membrane organization, as shown by the similar modulation of membrane properties observed in both *ΔrocS* and *rocS*_G155E_ cells. These data suggest that the *S. pneumoniae* compensates for either the absence of RocS or for its altered membrane‐targeting mechanism, reinforcing the functional coupling between the RocS kink‐helix motif and membrane homeostasis.

**FIGURE 8:**
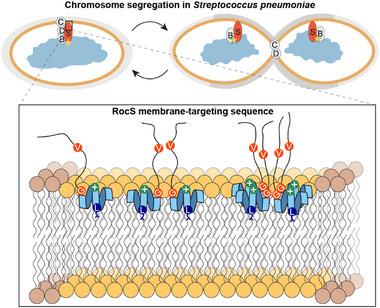
Mechanistic model of RocS MTS association with the membrane. Upper panel: Role of *Streptococcus pneumoniae* RocS (S) in chromosome segregation. ParB (B) represents the main molecular partner during cell division [2] and the yellow star indicates the *parS* binding site of ParB in the OriC region. Lower panel: Focus on the membrane‐targeting sequence (MTS). RocS MTS associates with thinner lipid nanodomains and assembles into oligomeric foci. The C‐terminal amphipathic helix (light blue) inserts at the membrane surface, preceded by the kink at Gly155 (orange G) and the flexible Val (orange V). The central Leu (blue L) might extend toward the hydrophobic membrane region, while the positively charged residue remains at the membrane surface.

Altogether, we reveal that the conserved RocS MTS adopts a kink‐helix conformation which interacts strongly with lipid bilayers in fluid membrane regions, playing a key role in membrane association and correct protein function (Figure 8). Notably, the cell division site positioning protein MinD, which is widespread among bacterial species including *E. coli* and *B. subtilis*, also harbors a similar conserved MTS (Figure 1C) [52]. Beyond bacteria, the presence of related sequences across all domains of life, suggest that the kink‐helix motif represents a fundamental structural module mediating specific lipid interactions and spatial regulation in peripheral membrane proteins. These findings pave the way for future studies on the molecular mechanisms by which lipid‐protein interplay governs bacterial cell division and other fundamental cellular processes.

## 4 Materials and Methods

### 4.1 Amino Acid Sequence Alignment

The amino acid sequences of RocS from *Streptococcus pneumoniae* (accession code: Q8DQ15) and MinD from *Escherichia coli* strain K12 (P0AEZ3), *Salmonella enterica* subsp. enterica serovar Typhimurium str. LT2 (Q8ZP10), *Yersinia pestis* CO‐92 (A0A5P8YEZ0), *Vibrio cholerae* M1526 (A0A5Q6PMH8), *Pseudomonas aeruginosa* PAO1 (Q9HYZ6), *Neisseria gonorrhoeae* CH811 (Q9AG19), *Xylella fastidiosa* strain M23 (B2I966), *Shewanella* sp (A0A9E6B3T8), *Listeria monocytogenes* CFSAN072502 (A0A823IWG3), *Clostridium ultunense* strain Esp (M1Z6E7), *Bacillus subtilis* 168 (Q01464), *Aquifex aeolicus* VF5 (O67033), *Methanocaldococcus jannaschii* strain ATCC43067 (Q57967), *Archaeoglobus fulgidus* SpSt‐87 (A0A7C3RCL2), *Pyrococcus furiosus* strain ATCC 43587 (A0A5C0XTN6), *Borrelia maritima* CA690 (A0A5J6W9N7), *Treponema pallidum* Nichols (Q56340), *Arabidopsis thaliana* cv. Columbia (Q9MBA2) and *Oryza sativa* subsp. Japonica (Q0DF98) were retrieved from UniProt database. More details on the sequence alignment can be found in the Materials and Methods paragraph of the *Supporting Information*.

### 4.2 Reconstitution of MTS Peptides into Membrane Vesicles

The synthetic MTS peptides (Ac‐AKEEVQATKKGFFARLFGGK‐OH), (Ac‐AKEEVQATKKGFFARLFG‐OH) and the selectively isotopically labeled MTS peptide (Ac‐AKEEV(13C5,15N)QATKKG(13C2,15N)FFARL(13C6,15N)FGGK‐OH) were purchased from GeneCust with a purity greater than 95% and N‐terminal acetylation.1‐palmitoyl‐2‐oleoyl‐glycero‐3‐phosphocholine (POPC), 1‐palmitoyl‐d31‐2‐oleoyl‐sn‐glycero‐3‐phosphocholine (POPC‐d31), 1‐palmitoyl‐2‐oleoyl‐sn‐glycero‐3‐phosphate (POPA), 1‐palmitoyl‐2‐oleoyl‐sn‐glycero‐3‐phospho‐(1'‐rac‐glycerol) (POPG), and cardiolipin from bovine heart (CL) were obtained from Avanti Polar Lipids, Inc. (USA). To prepare the samples, synthetic MTS peptides were dissolved together with lipids in a chloroform/methanol mixture (2:1, v/v), ensuring a peptide‐to‐lipid molar ratio of 1:20. The organic solvents were removed using an air stream, followed by hydration and subsequent lyophilization overnight. The resulting lipid‐peptide powder was then resuspended in 100 µl 25 mM Tris‐HCl pH 7.5, 150 mM NaCl and 5% glycerol, and homogenized through three cycles of vortexing, rapid freezing in liquid nitrogen (‐196°C, 1 min), and thawing in a 60°C water bath for 10 min. The same protocol was followed for the control sample, prepared under identical buffer conditions but without the peptide. All proteoliposome solutions appeared turbid, suggesting a particle size of approximately 0.1 – 1 µm. For MAS ssNMR experiments, the samples were ultracentrifuged at 42,000 rpm for 1 hour at 4°C using a TLA 120.1 rotor (Beckman Coulter) to pellet the proteoliposomes.

### 4.3 MAS Solid‐State NMR

MAS solid‐state NMR experiments were performed on a 600 MHz ^1^H Larmor frequency spectrometer (Bruker Biospin) using a 4 mm triple resonance HCN MAS probes. All magic‐angle spinning (MAS) ssNMR experiments were performed at a spin rate of 11 kHz. The sample temperature was estimated from the ^1^H signal of water according to the DSS (sodium 2,2‐dimethyl‐ 2‐silapentane‐5‐sulfonate) signal used as an internal reference [20]. SPINAL‐64 ^1^H decoupling power during acquisition of 83 kHz was used [53] (additional experimental parameters were listed in the Supporting Information Table S4). For the 2D ^13^C‐^13^C PDSD (proton‐driven spin diffusion), a ramped CP with 1 ms contact time was used during ^1^H‐^13^C cross‐polarization (CP), followed by a ^13^C‐^13^C mixing time of 50 or 150 ms. The 2D ^13^C‐^13^C DP‐PDSD (Direct Polarization proton‐driven spin diffusion) was recorded with a mixing time of 150 ms. In the 2D ^1^H‐^13^C heteronuclear correlation (HETCOR), the ^1^H excitation was followed by a ^1^H‐^13^C cross‐polarization with 0.75 ms contact time. For the extended HETCOR [27, 28, 29, 31]: following ^1^H excitation, a ^1^H T_2_ filter with a total echo period of 2×2.18 ms was applied, during which simultaneous 180° pulses on ^1^H and ^13^C channels were implemented. This T_2_ filter effectively suppresses rigid protein ^1^H magnetization, selectively retaining signals from mobile water and lipid protons. Subsequently, the selected water and lipid ^1^H magnetization was transferred to the protein via chemical exchange and spin diffusion during a z‐filter mixing period. The resulting protein ^1^H magnetization was then cross polarized to ^13^C nuclei for detection. No ^13^C signal was observed at a 0 ms ^1^H mixing time, confirming the effective suppression of initial protein ^1^H magnetization by the T_2_ filter. At a 100 ms mixing time, the resulting ^13^C spectra resembled those obtained by conventional cross‐polarization (CP), with approximately half the intensity, indicating equilibration between water and protein ^1^H magnetization by 100 ms. All MAS ssNMR spectra were processed and analyzed using Bruker Topspin 4.0 software (Bruker Biospin) and CCPNMR Analysis [54].

### 4.4 ^31^P and ^2^H Wide‐Line Solid‐State NMR Spectroscopy

For ^2^H static ssNMR, a static phase‐cycled quadrupolar spin echo pulse sequence was applied at the ^2^H frequency of 76.8 MHz on a Bruker Avance III 500 MHz NMR spectrometer with a 4 mm double resonance HX probe. The 90° pulse length was 3.5 µs with the interpulse delay of 50 µs having 2 s of recycle delay. The spectral window was fixed at 500 kHz and the spectra were recorded with 2816 number of scans. The spectra were acquired at various temperatures ranging from 263 K to 303 K. The sample was held at the desired temperature for 20 min prior to signal acquisition. The spectra were processed with Topspin 4.0.6 (Bruker) and a line broadening factor of 400Hz was applied prior to Fourier transformation. NMR Depaker (provided by Dr. Sébastien Buchoux) was used to compute first order spectral moments M1 and and were simulated on NMR‐099 (provided by Arnaud Grélard) to get local order parameters |2∗S_CD_| along the acyl chains of POPC‐d31 [32, 34].

The ^31^P NMR experiments were performed on 400 MHz (9.4 T) Bruker Avance III HD spectrometer. The spectra were recorded with static Hahn spin echo sequence for ^31^P frequency, with 1024 scans, with a 90° pulse of 8 µs, 40 µs delay, 5 s a recycle delay and the spectral width was set to 64 kHz. All spectra were processed with TopSpin 4.0.6 (Bruker), the free induction decay was processed using 50 Hz of line broadening [35].

### 4.5 Molecular Dynamics

Molecular dynamics simulations were performed using the GROMACS software [55] (version 2023.3). The initial model of the RocS C‐terminal helix fragment was computed with AlphaFold3 [12] using the sequence AKEEVQATKKGFFARLFGGK. The resulting α‐helical peptide was processed through the OPM webserver [56] and positioned ∼10 Å above the upper leaflet to prevent initial peptide‐membrane interactions. System preparation was achieved through CHARMM‐GUI with the Input Generator module and Bilayer Builder workflow [57, 58], taking the membrane‐oriented peptide as input. A homogeneous bilayer was prepared with POPC (0.65), POPG (0.25), and cardiolipin TLCL2 (0.10). Simulations were done in triplicate using the CHARMM36m force field and TIP3P water model. The system was solvated in a rectangular box with an appropriate buffer distance from the peptide and 50 mM KCl was added to neutralize the system. Energy minimization was achieved in GROMACS using the steepest descent algorithm with position restraints on the backbone, side chains, and lipids until a stable configuration was obtained. Equilibration was performed in multiple stages under NPT conditions for a total of 2.25 ns with gradually decreasing restraints. Particle Mesh Ewald was used for long‐range electrostatics, a 1.2 nm cutoff was used for van der Waals and Coulomb interactions, and hydrogen bonds were constrained using LINCS. Production simulations were performed under NPT conditions for 5 µs at 308 K and 1 bar using semi‐isotropic pressure coupling, with a 2 fs time step and the Verlet cutoff scheme. Trajectories were fitted to the protein to remove overall translation and rotation. Helix bending was quantified using a vector‐based approach with gmx bundle, defining vectors along the Cα atoms of the top (residues 3‐10) and bottom (residues 11‐18) segments and calculating the bending angle as θ = arccos[(v1⋅v2)/(∥v1∥∥v2∥)], then converted to degrees, yielding a helix bending angle for each frame.

### 4.6 Atomic Force Microscopy AFM

Supported lipid bilayers were prepared for AFM imaging experiments by vesicle fusion on mica surfaces. To obtain SUVs (small unilamellar vesicles), an MLV suspension of POPC/POPG/POPA/CL (65/15/15/5) was sonicated until reaching clarity. Freshly cleaved mica was heated to 75°C on a heating plate, 10 µL of CaCl_2_ 2 mM was deposited on the mica surface, immediately followed by the deposition of 70 µL of the SUV suspension. The system was left to incubate on the hot plate for 30 min to 1 hour, in a humid atmosphere and with regular addition of buffer to avoid drying. After incubation, the sample was allowed to cool down to room temperature, followed by rinsing with buffer (100 µL of buffer, 5 to 10 times). The bilayer was then imaged in buffer using the PFQNM mode (Peak Force Quantitative NanoMechanics), with a 1 kHz peak force frequency, 30 nm amplitude, and between 300 and 500 pN of force. MSNL‐E probes were used, with a nominal spring constant of 0.2 N/m and a nominal radius of 2 nm.

### 4.7 Summary of In Vitro Experimental Conditions

Table S7 summarizes all experimental conditions used for the in vitro experiments (Figures 2, 3, 4, 5, 6).

### 4.8 Strain Construction

The strains and primers used in this study were detailed respectively in Supporting Information Tables S5 and S6. Strains of *S. pneumoniae* were derived from R800 rpsL1 (resistant to streptomycin) and transformed with PCR products of interest as previously described [4]. Briefly, to remove the kanR‐rpsL cassette, we first amplified the upstream fragment with the primer pair 1113‐5484 and the downstream fragment with the primer pair 5483‐1116 from Spn5 and Spn1024, respectively, for the construction of *rocS_G155E_* and *gfp‐rocS_G155E_*. The fragments were fused and transformed into the *ΔrocS*::Janus strain. Bacteria were cultivated in C + Y medium at 37°C without agitation, and competence was induced at around OD_550_ = 0.1 by the addition of the synthetic competence stimulating peptide 1 (CSP1) [59]. DNA was then mixed with competent cells and incubated at 37°C for 30 min before plating dilutions of the transformed bacteria mixed in THY‐agar supplemented with 3 % of defibrinated horse blood. After 2 h of incubation at 37°C, an additional THY‐agar layer supplemented with streptomycin 400 µg/mL was poured and the cells were left to incubate overnight. All strains were verified by PCR, antibiotic sensitivity testing, and locus sequencing.

### 4.9 Preparation of *S. pneumoniae* Crude Extracts and Immunoblot Analysis

Pneumococcal cells were first grown in C + Y medium until they reached early log phase (OD_550nm_ = 0.1–0.2). They were then normalized at OD_550nm_ = 0.001 in the corresponding medium and grown again until they reached an OD_550nm_ = 0.1, and then centrifugated at 5,000 g for 5 min. Pellets were resuspended in a lysis buffer (Tris‐HCl 10 mM pH 8, EDTA 1 mM supplemented with 1:100 protease inhibitors cocktail). After cell disruption by sonication, non‐lysed cells were discarded by centrifugation and crude extracts were normalized, analyzed by SDS‐PAGE and transferred onto an immobilon‐P membrane (Millipore). Primary antibodies were used at 1:10,000 (anti‐GFP, AmsBio) in TBST‐BSA 1% and 1:250,000 (anti‐enolase [60] in TBST‐BSA 5%. The goat anti‐mouse secondary antibody HRP conjugate was used at 1:5,000 (Biorad) in TBST‐BSA 1%. The revelation was performed with Chemiluminescent SuperSignal West Pico PLUS kit (ThermoScientific) and imaged with Fusion Fx7 camera (Vilber Lourmat).

### 4.10 Microscopy and Image Analyses

After cells preculture reaching an OD_550_ up to 0.2‐0.3, cells were diluted to OD_550_ = 0.001 and grown at 37°C in C + Y medium to OD_550_ = 0.1. Then 0.8 µl was spotted onto a 1 % agarose/C + Y pad. When appropriated, pads were supplemented with FM4‐64 at 10 µg/mL (Sigma‐Aldrich, Ref. T3166) or DAPI at 2 µg/mL. Visualization was performed using a Nikon TiE microscope fitted with an Orca‐CMOS Flash4 V2 camera with a 100 × 1.45 objective. Images were collected using NIS‐Elements (Nikon). Image analysis was performed using MicrobeJ 5.13p ‐ beta version [61]. For the quantification of the chromosome segregation defects, cells were classified according to their DAPI profile [2, 4]. Statistical analyses were done with GraphPad Prism version 10.5.0. The tests used were indicated in figure legends. In addition, the Shapiro–Wilk normality test was used for all tests.

## Author Contributions

Conceptualization, methodology, investigation, visualization: A.A.M., E.M., C.L., A.K.P., M.L., Z.X., N.E.M., F.M., E.J.D., C.F., C.G., B.H.; Supervision: C.G., B.H.; Writing – review & editing: all authors.

## Conflicts of Interest

The authors declare no conflicts of interest.

## Supporting information




**Supporting File**: advs73724‐sup‐0001‐SuppMat.docx.

## Data Availability

The data that support the findings of this study are available in the Supplementary Information or upon reasonable request from the corresponding authors.
