# Supplementary material for: Structural Basis of the Membrane Association by the Conserved RocS Membrane‐Targeting Sequence in Streptococcus
Source: Adv Sci (Weinh). 2026 Jan 15;13(17):e21132. doi: 10.1002/advs.202521132 (PMC13042908; doi:10.1002/advs.202521132)
Supplement: Supplementary file 1 — Supporting File: advs73724‐sup‐0001‐SuppMat.docx. [file ADVS-13-e21132-s001.docx]

**Supporting Information**

**A kink-helix motif regulates nanodomain association of the chromosome segregation factor RocS in *Streptococcus pneumoniae***

**Authors**

Ana Álvarez-Mena^1#^, Estelle Morvan^2#^, Clara Lambert^3^, Anagha Kallisseri Parambil^1^, Martin Lefeuvre^1^, Zeren Xu^1^, Nadia El Mammeri^1^, Florian Malard^4^, Erick Dufourc^1^, Cécile Feuillie^1^, Christophe Grangeasse^3^*, Birgit Habenstein^1^*

**Affiliations**

1 Univ. Bordeaux, CNRS, Bordeaux INP, CBMN, UMR 5248, Pessac, France

2 Univ. Bordeaux, CNRS, Inserm, IECB, UAR3033, US01, Pessac, France

3 Université Claude Bernard Lyon 1, CNRS, MMSB, UMR 5086, Lyon, France.

4 Univ. Bordeaux, CNRS, INSERM, ARNA, UMR 5320, U1212, F-33000 Bordeaux, France

^#^ these authors contributed equally

*corresponding author: C. Grangeasse christophe.grangeasse@cnrs.fr ; B. Habenstein [b.habenstein@cbmn.u-bordeaux.fr](mailto:b.habenstein@cbmn.u-bordeaux.fr)


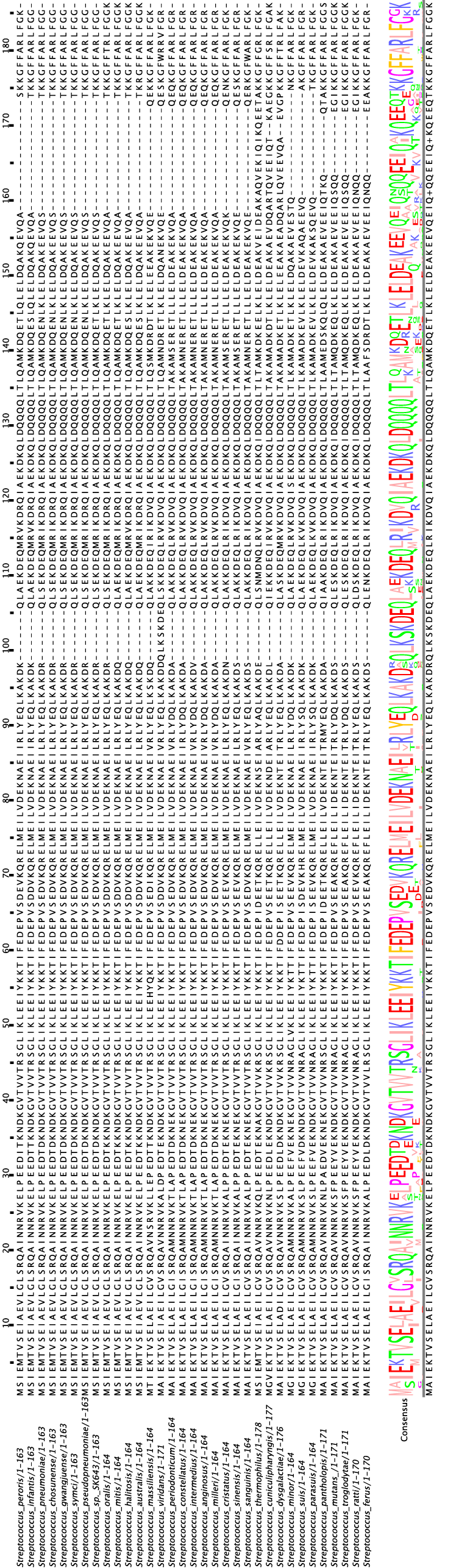


**Supporting Fig. S1: Amino acid sequence alignment of homologous proteins from different *Streptococcus* species.** Multiple sequence alignment was performed using the *S. pneumoniae* protein as a reference. The degree of conservation of the residues across species is illustrated at the bottom.


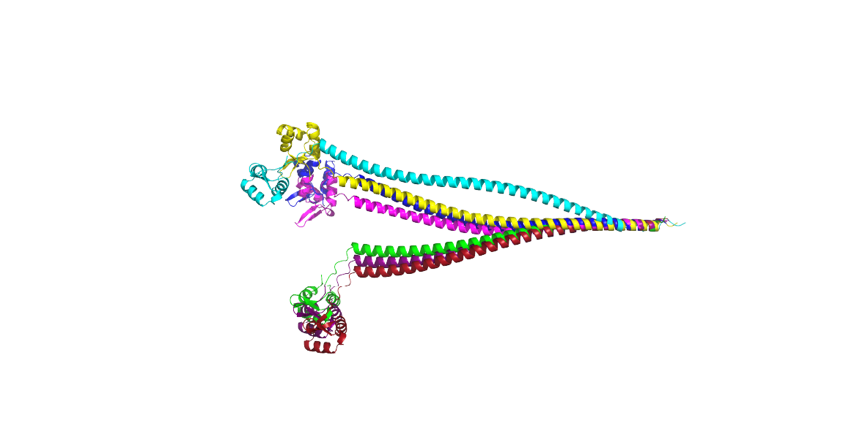


**Supporting Fig. S2: Structural alignment of full-length RocS homologs from relevant *Streptococcus* species.** Homologous regions from *S. gallolyticus* (cyan), *S. anginosus* (purple), *S. sanguinis* (green), *S. suis* (brown), *S. mitis* (magenta), and *S. mutants* (yellow) were aligned using the C-terminal region of *S. pneumoniae* (residues A145-G163, blue) as a reference.


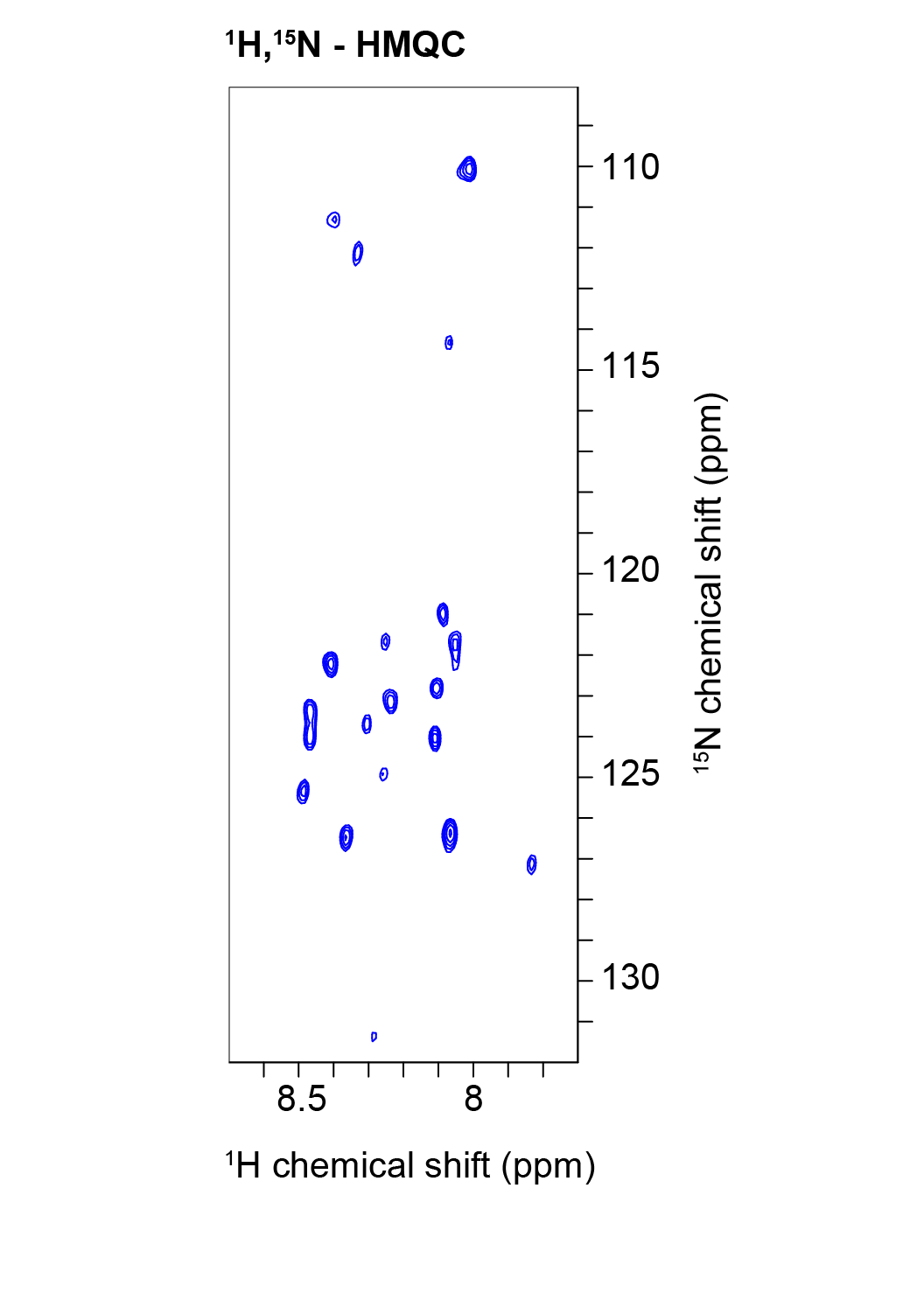


**Supporting Fig. S3:** 2D ^1^H-^15^N SOFAST-HMQC spectrum of the natural abundance short RocS MTS peptide (AKEEVQATKKGFFARLFG) acquired by solution NMR at 298 K, showing well-dispersed cross-peaks consistent with a folded conformation. The experiment was performed on an 800 MHz spectrometer using a cryoprobe.


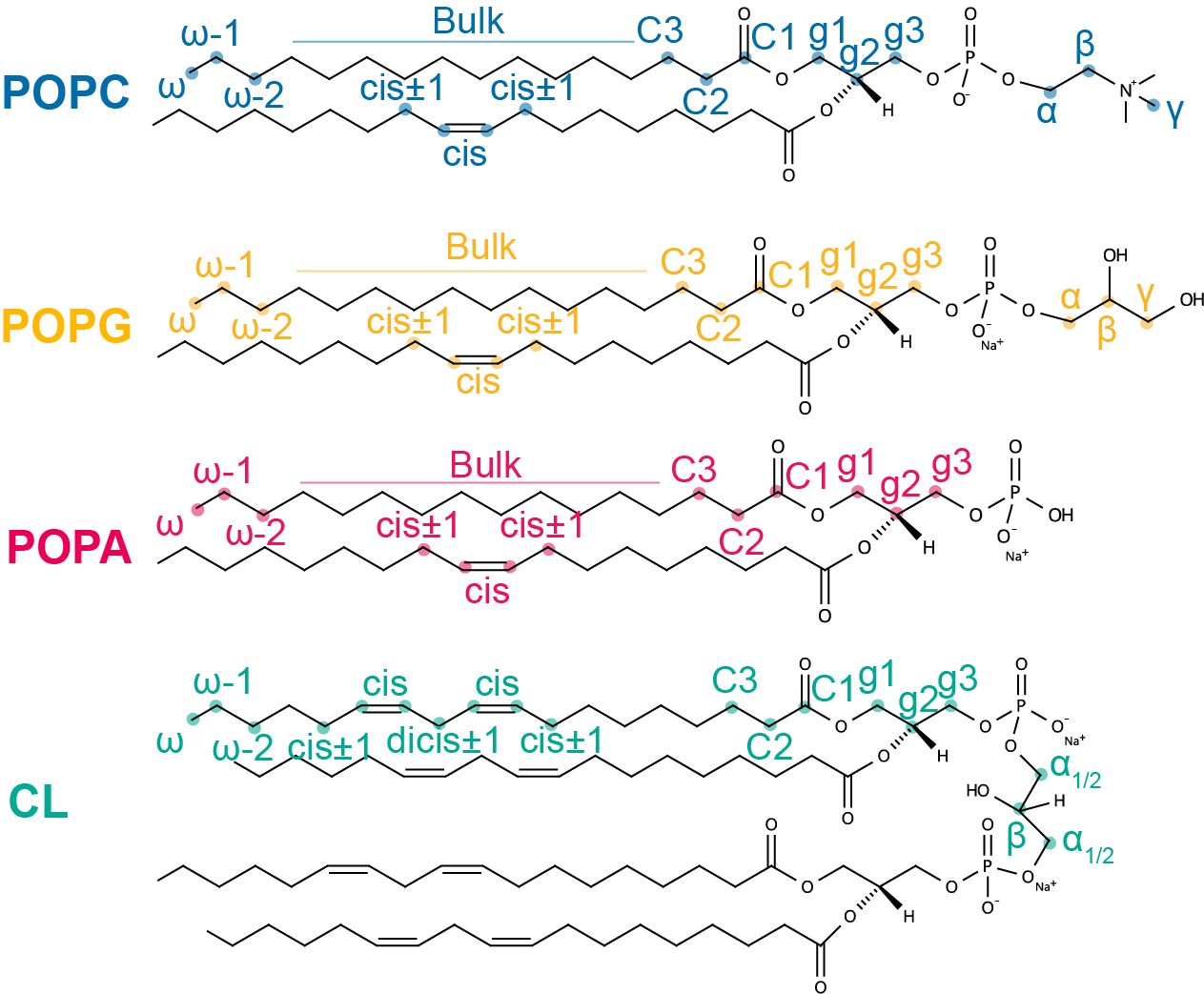


**Supporting Fig. S4:** Chemical structures of the lipids used in the preparation of multilamellar vesicles (MLVs): POPC, POPG, POPA, and cardiolipin (CL), mixed at a 65:15:15:5 molar ratio. Individual atoms are labeled to facilitate interpretation of NMR chemical shifts.


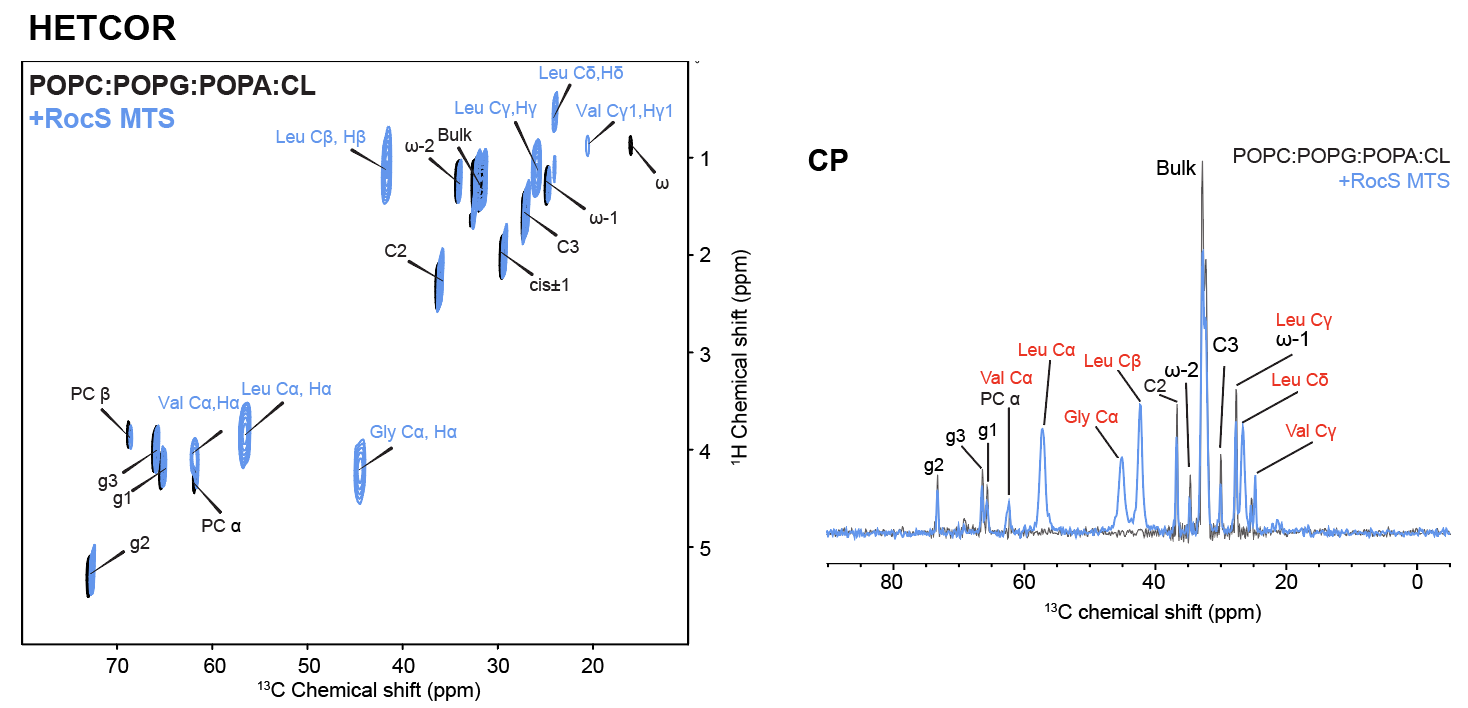


**Supporting Fig. S5:** Left: Superimposed ^1^H-^13^C HETCOR spectra acquired at 30°C (292 K indicated temp.) on a 600 MHz spectrometer with a MAS spinning frequency of 11 kHz. The spectrum of POPC:POPG:POPA:CL (65:15:15:5) MLVs alone (black) is compared to that of the same lipid mixture in the presence of the isotopically labeled MTS RocS peptide (light blue) at a peptide-to-protein lipid ratio (1:20). Chemical shifts detected in both conditions are labeled in black, while those observed only in the presence of the MTS RocS peptide are labeled in light blue. Right: 1D ^13^C cross-polarization (CP) spectra are shown for each experimental condition. Chemical shifts appearing exclusively in the presence of the MTS RocS peptide are labeled in red.


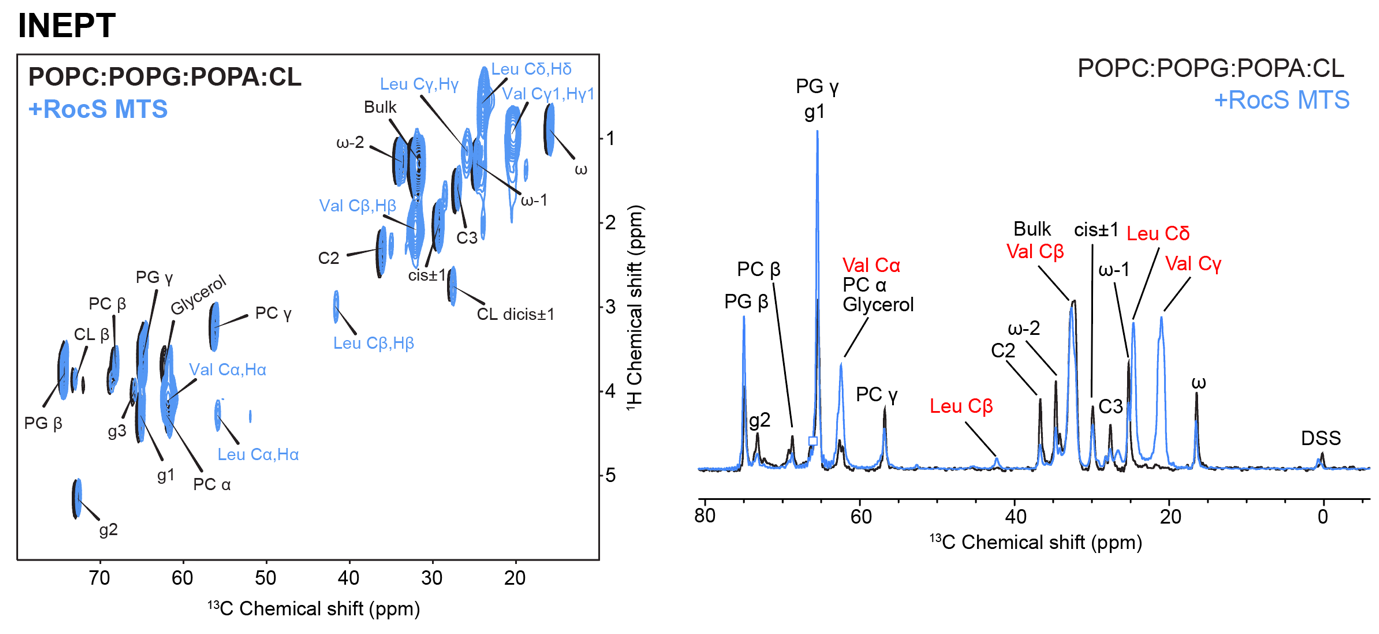


**Supporting Fig. S6:** Left: Superimposed ^1^H-^13^C INEPT spectra acquired at 30 °C (292 K indicated temp.) on a 600 MHz spectrometer with a MAS frequency of 11 kHz. The spectrum of POPC:POPG:POPA:CL (65:15:15:5) MLVs alone (black) is compared to that of the same lipid mixture in the presence of the isotopically labeled MTS RocS peptide (light blue) at a peptide-to-protein lipid ratio (1:20). Chemical shifts detected in both conditions are labeled in black, while those observed only in the presence of the MTS RocS peptide are labeled in light blue. Right: The corresponding 1D ^13^C INEPT spectra are shown for each experimental condition. Chemical shifts appearing exclusively in the presence of the MTS RocS peptide are labeled in red.


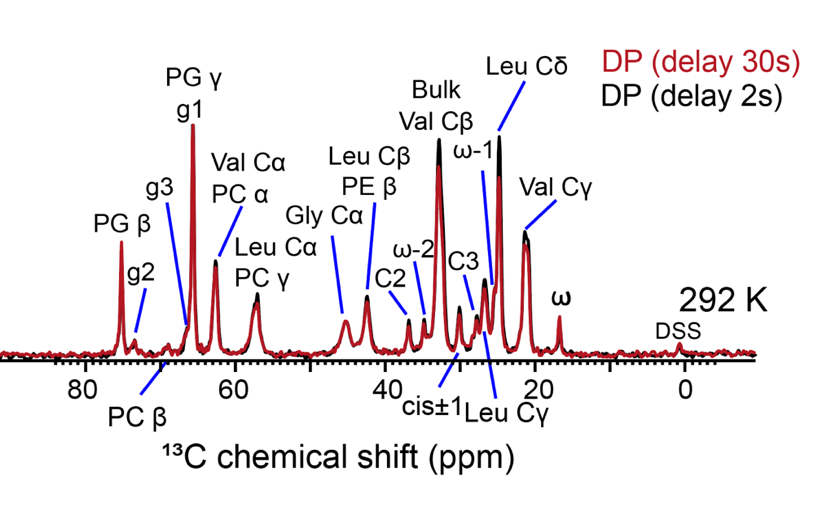


**Supporting Fig. S7:** 1D ^13^C DP spectrum of the selectively labeled MTS RocS peptide reconstituted into POPC:POPG:POPA:CL (65:15:15:5) MLVs. The peptide was incorporated at a peptide-to-lipid molar ratio of 1:20. The spectrum was recorded at 30°C (292 K indicated temp.) on a 600 MHz spectrometer with a MAS frequency of 11 kHz. The recycle delay was set to 30 sec for quasi-quantitative measurements and 2 sec to favor flexible moieties with shorter T_1_ relaxation times.

**
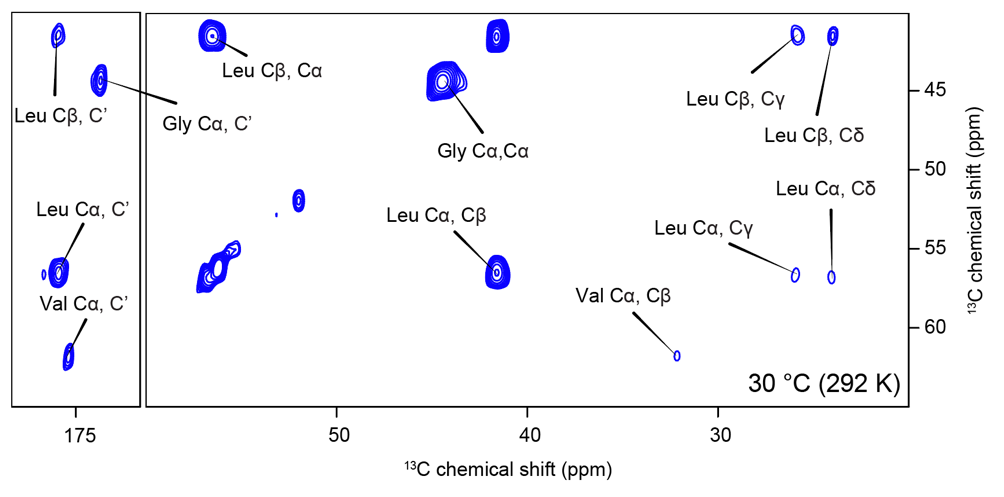
**

**Supporting Fig. S8:** 2D ^13^C-^13^C DP-PDSD spectrum of the selectively labeled MTS RocS peptide reconstituted into POPC:POPG:POPA:CL (65:15:15:5) MLVs. The peptide was incorporated at a peptide-to-lipid molar ratio of 1:20. The spectrum was recorded at 30°C (292 K indicated temp.) on a 600 MHz spectrometer with a MAS frequency of 11 kHz. Cross-peaks correspond to intra-residue contacts of the selectively labeled residues within the MTS RocS peptide.


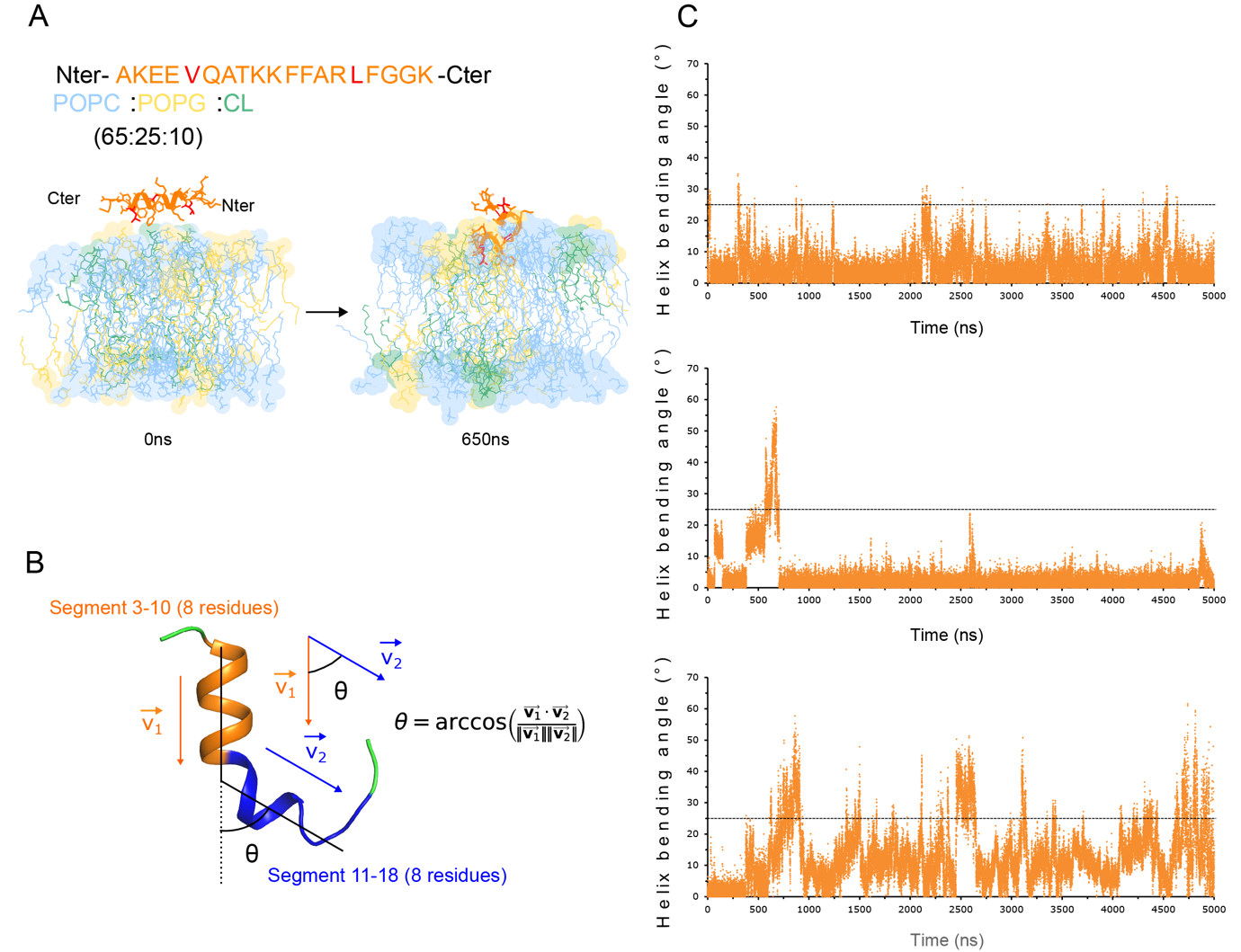


**Supporting Fig. S9:** All-atom molecular dynamics simulations using the GROMACS software and the CHARMM36m force field were performed in triplicate. The MTS peptide AKEEVQATKKGFFARLFGGK was placed at ~10Å distance from the POPC:POPG:CL (65:25:10 mol:mol) surface and the MTS-membrane interaction is monitored over 5 μs simulation. (A) Snapshots at 0 ns and 650 ns timepoints showing the insertion and the formation of a kink. ^13^C-labeled residues, experimentally assessed in the NMR sections, are highlighted in red. (B) Structural representation of the helix bending angle θ calculated as θ=arccos[(v1⋅v2)/(∥v1∥∥v2∥)], interpreted in (C) to demonstrate helix bending around kink residue Gly155.

**
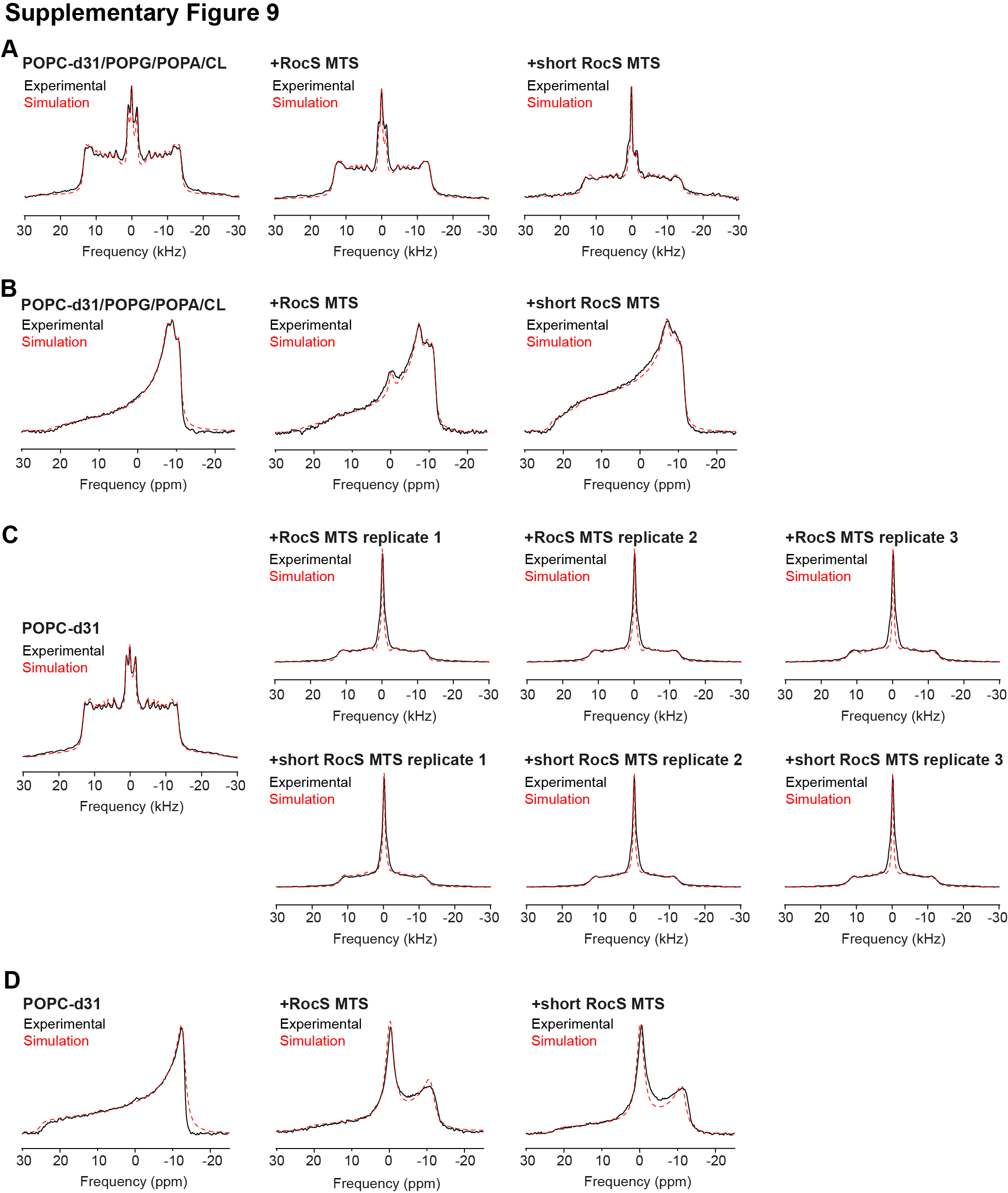
**

**Supporting Fig. S10:** Simulations of the ^2^H and ^31^P-detected wide-line ssNMR spectra. Simulated spectra (dashed line in red) are shown over the recorded spectra (black). **A, B** RocS MTS interactions with vesicles of POPC:POPG:POPA:CL composition doped with POPC-d31. **A** ^2^H-detected NMR spectra. **B** ^31^P-detected NMR spectra. The simulations show no marked difference between the CSA of the different lipids. Simulations include c/a ratio representing the ratio of an ellipsoid long axis to short axis[1] indicative of membrane deformation induced by the magnetic field and, here, reveal an impact of short RocS MTS which cancels the deformation seen on the complex membrane (1.4 membrane alone versus 1.0 membrane + short RocS MTS). **C, D** RocS MTS interactions with vesicles containing POPC, doped with POPC-d31; data represented in C and D are equivalent to those in A and B, respectively. Simulations have been performed as in Àlvarez-Mena et al. 2025.[2]^,^[3]^,^[4]

**
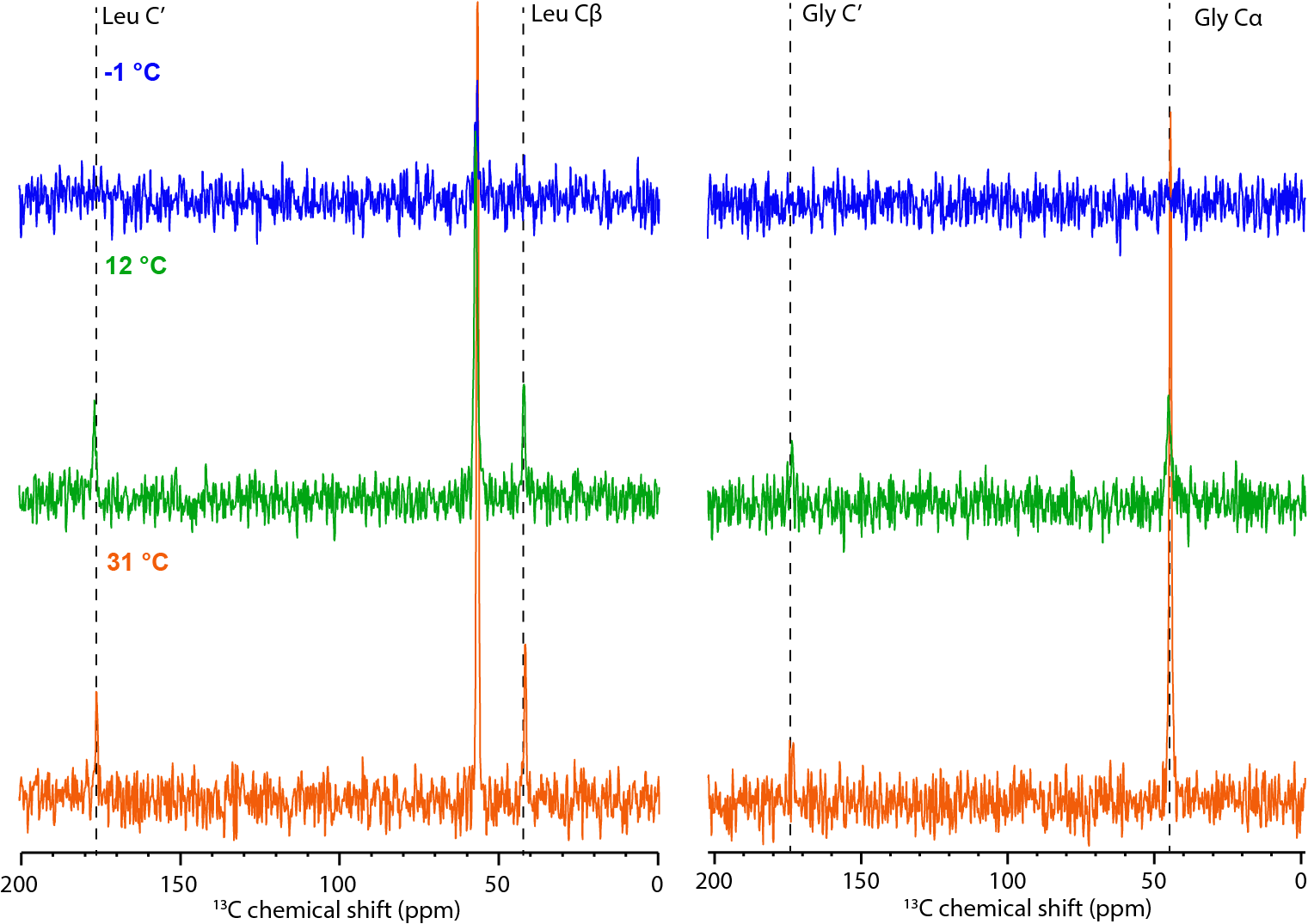
**

**Supporting Fig. S11:** 1D traces of the 2D PDSD of Figure 5 (main text). Left panel : trace centered around 57 ppm, on the Leu Cα position. Right panel : trace centered around 44.5 ppm, on the Leu Cα position.


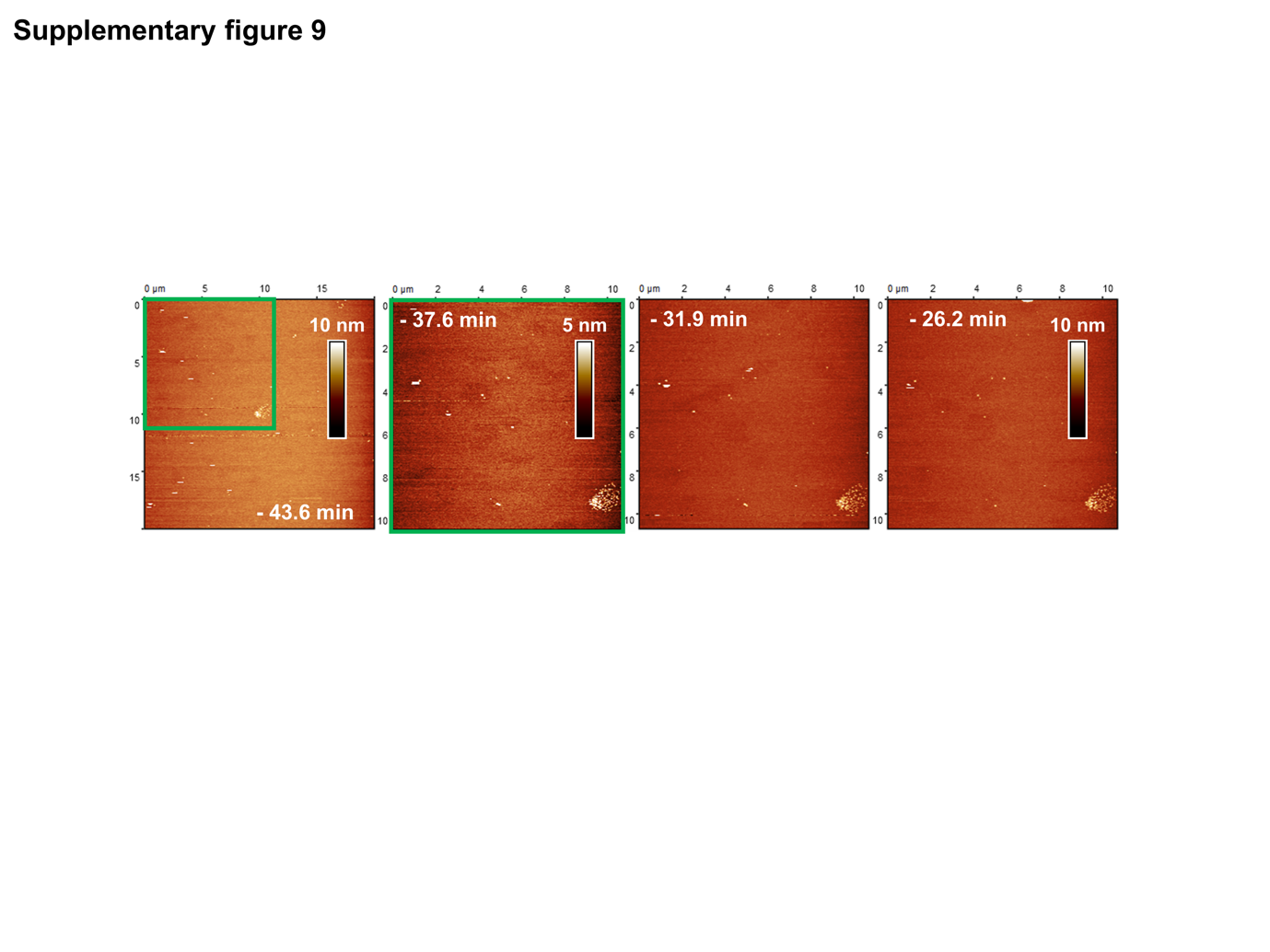


**Supporting Fig. S12**: Membrane stability over a 1-hour timescale. Timing indicates the time before peptide addition. The green square area is represented in the main Fig. 6 upon RocS MTS (including C-terminal Lys residue) addition.


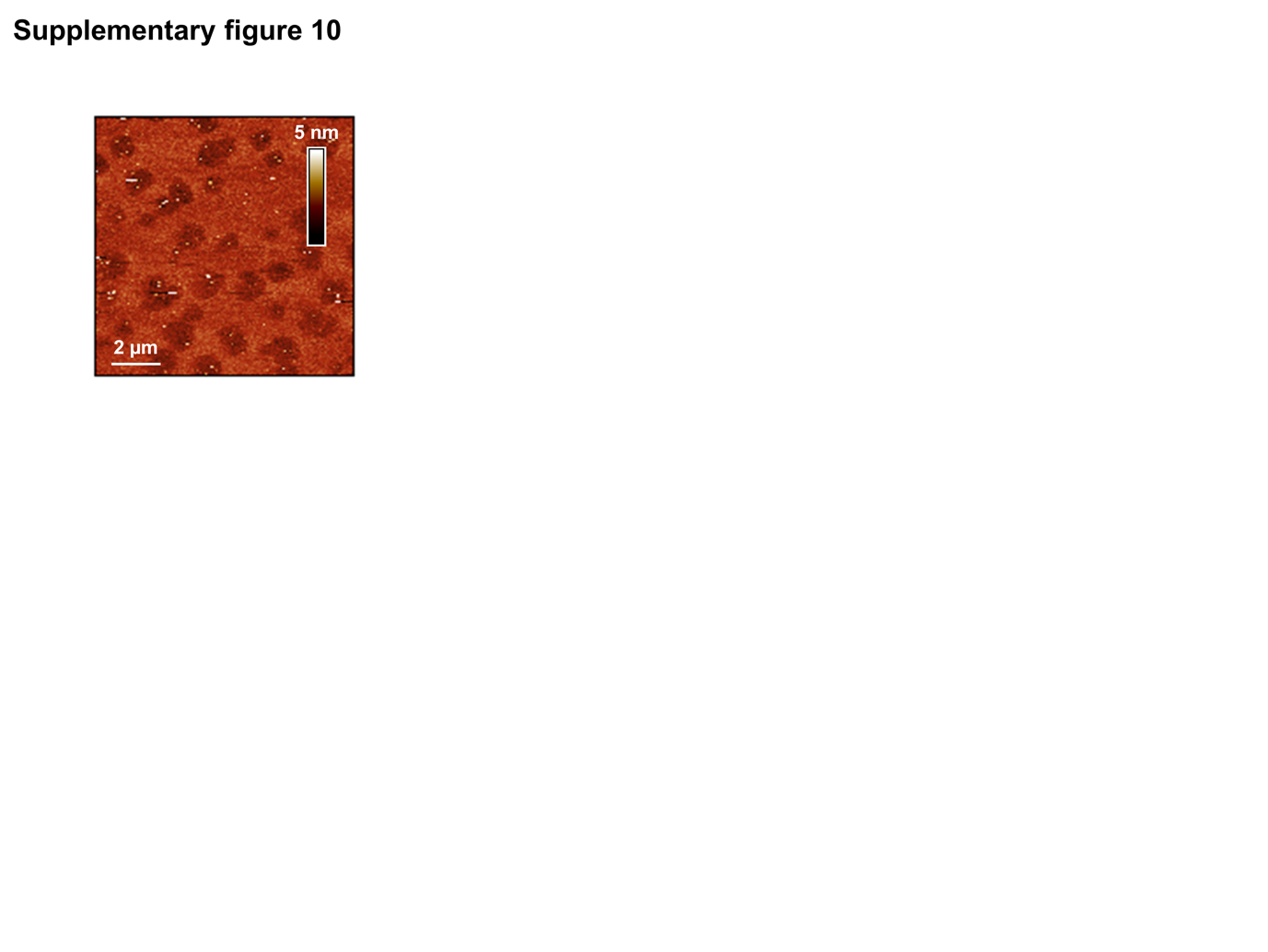


**Supporting Fig. S13:** Membrane nanodomains. Domain size (measured on ~ 12 domains) : Depth of 0.6 nm +/- 0.2 nm and width of 1.35 μm +/- 0.43 μm.


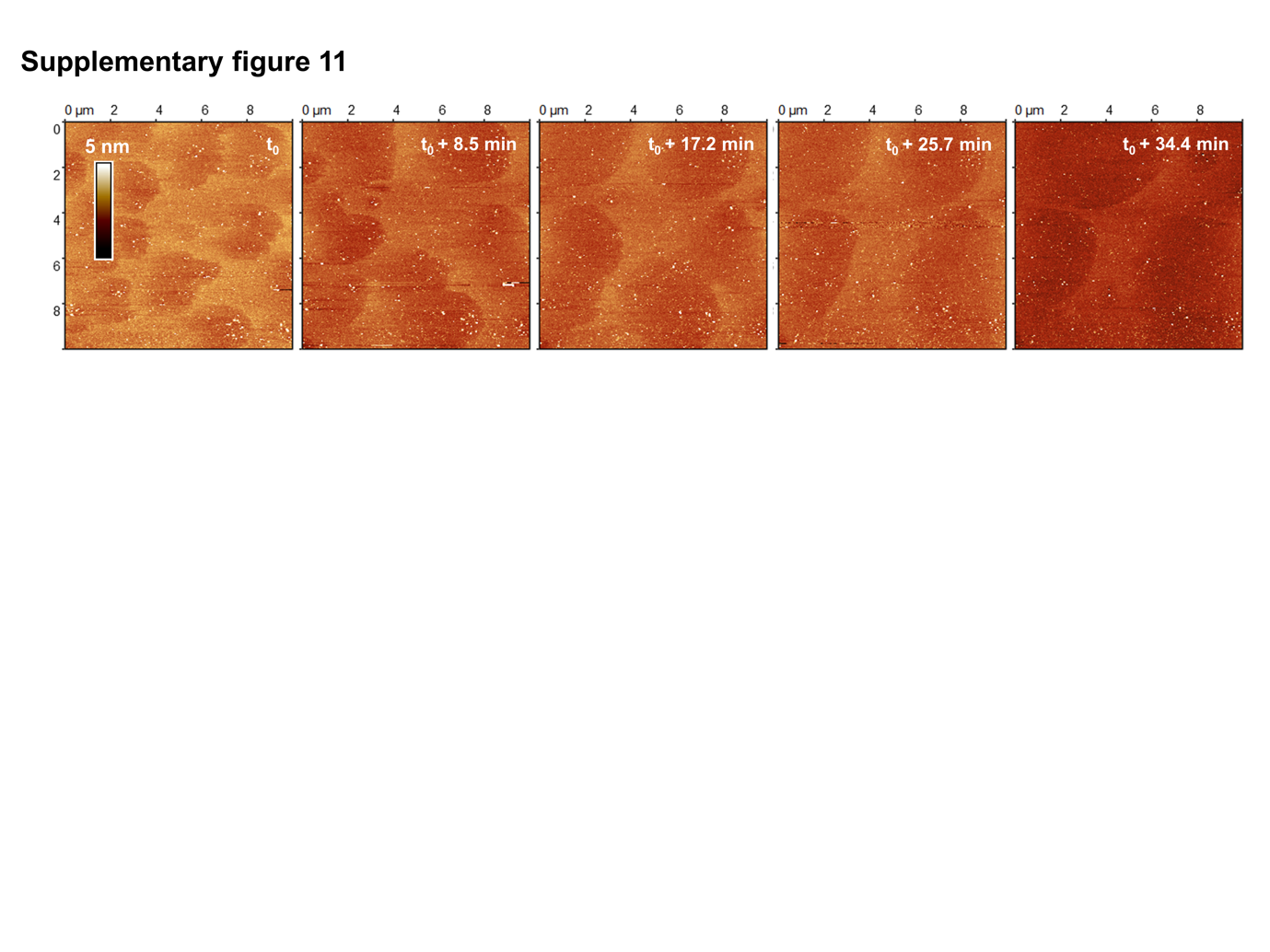


**Supporting Fig. S14:** Membrane remodelling without peptide addition over time; the nanodomains change in precise position and size over time.


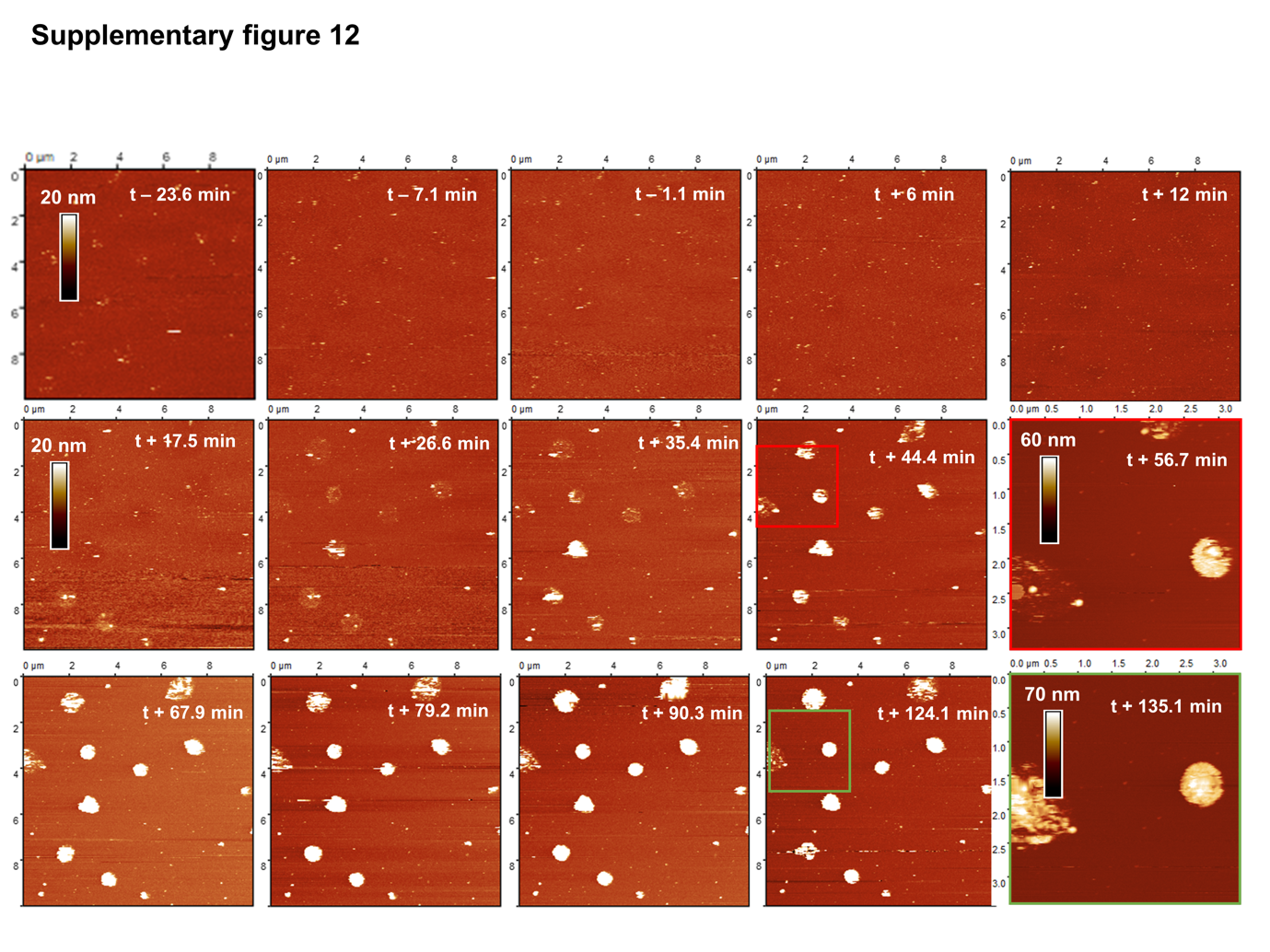


**Supporting Fig. S15:** AFM real time imaging of a POPC/POPG/POPA/CL bilayer before and after addition of 10 uM of short RocS MTS (lacking the C-terminal Lys). Selected images of the chronological sequence (red and green square) of the sequence are presented in higher detail after addition of the peptide.


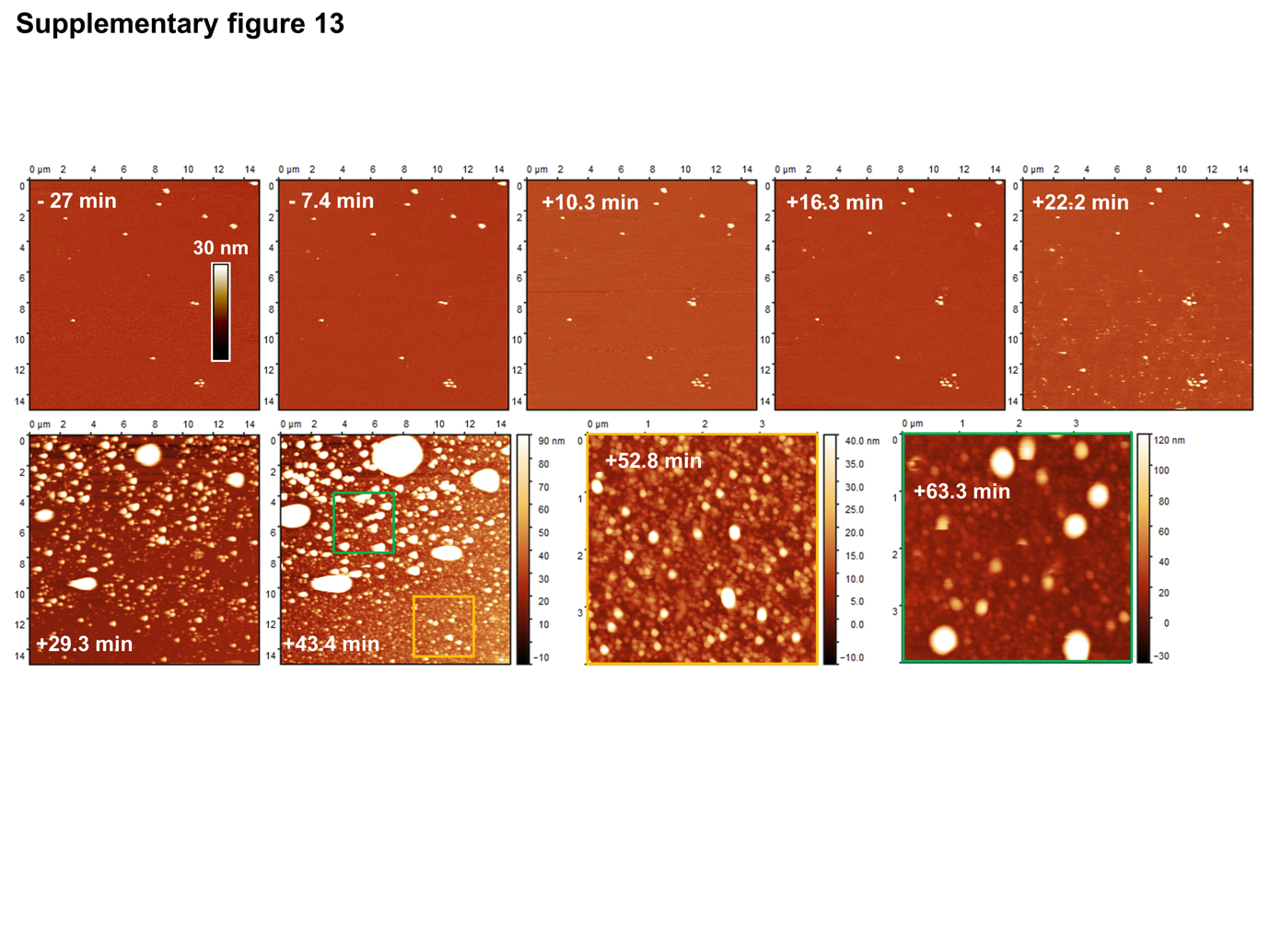


**Supporting Fig. S16:** AFM real time imaging of a POPC/POPG/POPA/CL bilayer before and after addition of 5 μM of RocS MTS (full peptide). Selected images (green and yellow squares) of the sequence are presented in higher detail after addition of the peptide.


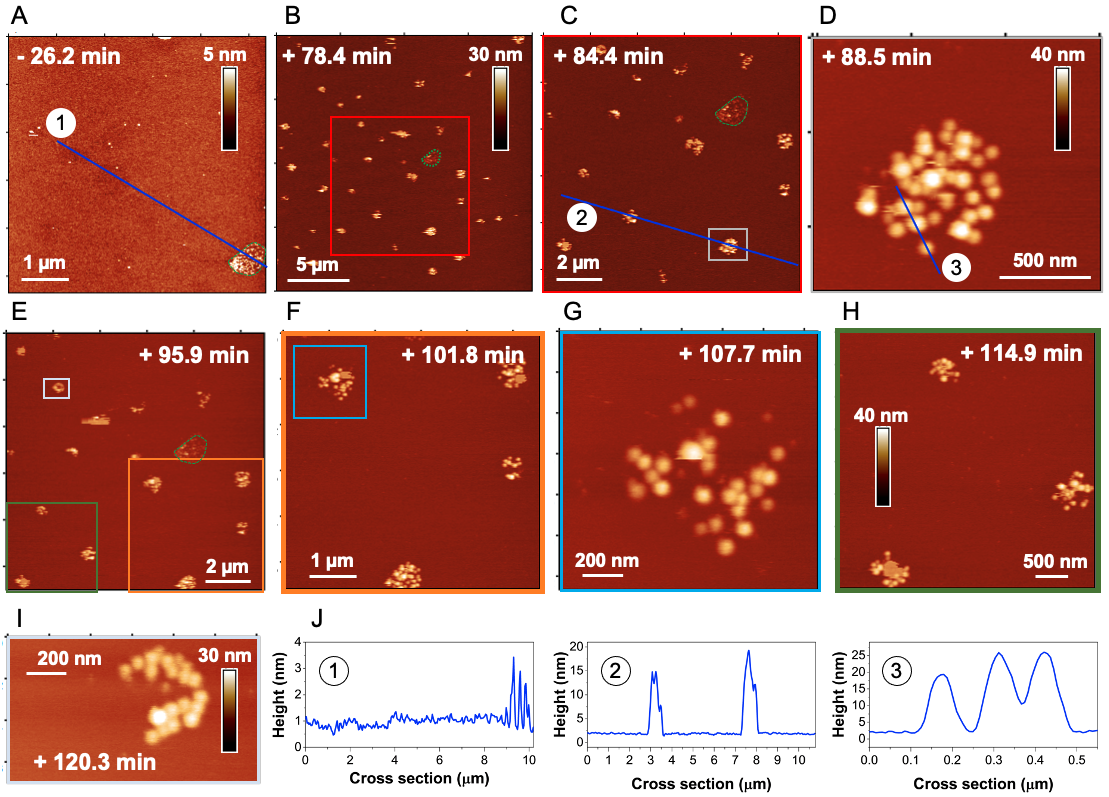


**Supporting Fig. S17:** AFM real-time imaging of the RocS MTS - membrane interaction (0.5 μM of RocS MTS, full peptide). Selected images of the sequence are presented chronologically, 26.2 min before addition of the peptide (A), and from 78.4 to 120 min after RocS MTS peptide addition (B - I). An initial defect in the SLB, highlighted by dashed green lines, serves as a landmark through time (visible on panels A, B, C, E). Cross-sections corresponding to the blue lines labeled 1, 2 and 3 J show the initial presence of lower domains in the membrane, that are eventually covered with protein clusters. Colored squares indicate the location of subsequent zoomed images (C corresponds to the red square in B; D corresponds to the grey square in C; F, G and I correspond to the orange, green and grey squares in E, respectively; G corresponds to the blue square in F).


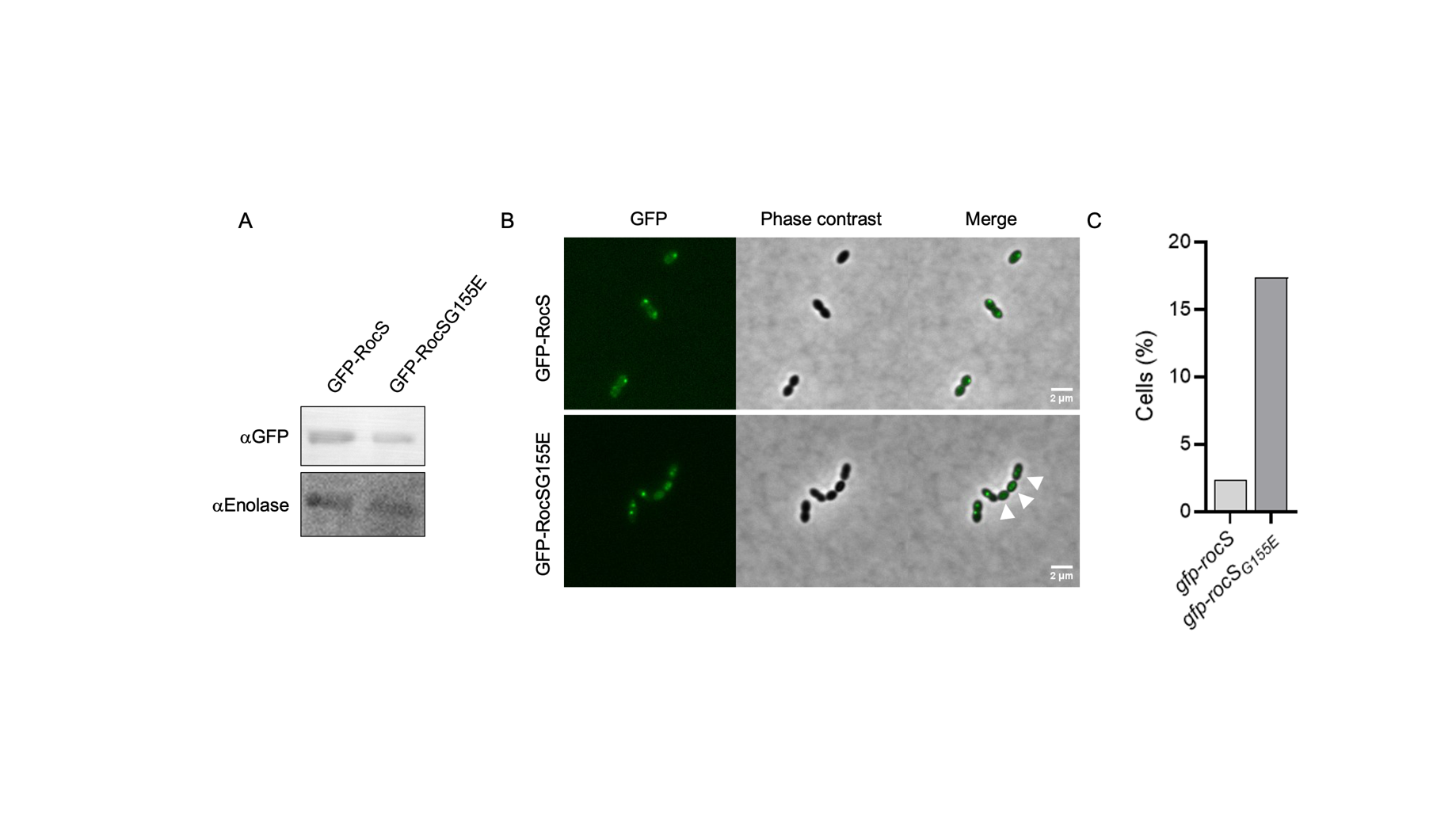


**Supporting Fig. S18:** Impact of the G155E mutation on RocS production and localization. (A). Western immunoblot of whole-cell lysates of rocS-gfp and rocSG155E-gfp strains with specific anti-GFP antibody and anti-enolase antibody as a loading control. (B). Images showing the localization of RocS-GFP and RocSG155E-GFP. Merged images between the GFP and the phase contrast channels are shown on the right. The arrowhead indicates cells with a cytoplasmic localization of RocS. Scale bars, 2 µm. (C) Percentage of cells with a cytoplasmic localization of RocS-GFP or RocSG155E-GFP.

**Supporting Table 1.** ^13^C chemical shifts (ppm) of the peptide Ac-AKEEV(^13^C_5_,^15^N)QATKKG(^13^C_2_,^15^N)FFARL(^13^C_6_,^15^N)FGGK-OH obtained from PDSD experiments at 264, 273, and 292 K with various mixing times. The peptide was reconstituted into a lipid mixture of POPC:POPA:POPG:CL (65:15:15:5) at a protein-to-lipid molar ratio of 1:20. The symbol ‘-‘ indicates unassigned carbon sites.

| **Amino acid type** | | **Carbon site (ppm)** | | | | | |
| --- | --- | --- | --- | --- | --- | --- | --- |
| **Gly** | | | | | | | |
| Temperature (K) | Experiment | CO | C**α** |  | | | |
| 264 | PDSD 50 ms | - | - |  |  |  |  |
| 273 | PDSD 50 ms | - | 44.432 |  |  |  |  |
| 292 | PDSD 50 ms | - | 44.382 |  |  |  |  |
|  | PDSD 150 ms | 173.106 | 44.569 |  |  |  |  |
|  | DP-PDSD 150 ms | 173.079 | 44.451 |  |  |  |  |
| **Leu** | | | | | | | |
| Temperature (K) | Experiment | CO | C**α** | C**β** | C**δ1** | C**δ2** | C**γ** |
| 264 | PDSD 50ms | - | - | - | - | - | - |
| 273 | PDSD 50ms | 176.350 | 56.890 | 41.398 | - | - | - |
| 292 | PDSD 50ms | 176.344 | 56.414 | 41.640 | - | - | - |
|  | PDSD 150 ms | 176.350 | 56.489 | 41.666 | 23.979 | 23.979 | 25.885 |
|  | DP-PDSD 150 ms | 176.356 | 56.512 | 41.599 | 23.989 | 23.989 | 25.840 |
| **Val** | | | | | | | |
| Temperature (K) | Experiment | CO | C**α** | C**β** | C**γ1** | C**γ2** |  |
| 264 | PDSD 50ms | - | - | - | - | - |  |
| 273 | PDSD 50 ms | - | - | - | - | - |  |
| 292 | PDSD 50 | - | - | - | - | - |  |
|  | PDSD 150 ms | - | - | - | - | - |  |
|  | DP-PDSD 150 ms | 175.564 | 61.823 | 32.146 | - | - |  |

**Supporting Table 2.** ^1^H and ^13^C chemical shifts (ppm) observed in 2D INEPT and HETCOR experiments at 292 K in POPC:POPA:POPG:CL (65:15:15:5) liposomes.

| Compound | Atom/Region | δ^1^H (ppm) | δ^13^C (ppm) | Experiment |
| --- | --- | --- | --- | --- |
| POPC:POPA:POPG:CL | **ω** | **0.876** | **15.995** | **INEPT, HETCOR** |
|  | **ω-1** | **1.307** | **24.980** | **INEPT, HETCOR** |
|  | **ω-2** | **1.265** | **34.355** | **INEPT, HETCOR** |
|  | **Bulk** | **1.301** | **32.157** | **INEPT, HETCOR** |
|  | **C1** | **-** | **-** | **-** |
|  | **C2** | **2.367** | **36.358** | **INEPT, HETCOR** |
|  | **C3** | **1.613** | **27.319** | **INEPT, HETCOR** |
|  | **g1** | **4.342** | **65.392** | **INEPT, HETCOR** |
|  | **g2** | **5.275** | **72.973** | **INEPT, HETCOR** |
|  | **g3** | **4.017** | **66.124** | **INEPT, HETCOR** |
|  | **cis±1** | **2.035** | **29.586** | **INEPT, HETCOR** |
| POPC | α | **4.306** | **61.990** | **INEPT, HETCOR** |
|  | β | **3.698** | **68.421** | **INEPT, HETCOR** |
|  | γ | **3.244** | **56.451** | **INEPT** |
| POPG | α | **-** | **-** | **-** |
|  | β | **3.750** | **74.622** | **INEPT** |
|  | γ | **3.565** | **65.061** | **INEPT** |
| CL | α | **-** | **-** | **-** |
|  | β | **3.863** | **73.299** | **INEPT** |
|  | **dicis±1** | **2.749** | **27.902** | **INEPT** |
| Glycerol | **C1/C3** | **3.700** | **62.275** | **INEPT** |
|  | **C2** | **-** | **-** | **-** |

**Supporting Table 3.** ^1^H and ^13^C chemical shifts (ppm) observed in 2D INEPT and HETCOR experiments at 292 K for the peptide Ac-AKEEV(^13^C_5_,^15^N)QATKKG(^13^C_2_,^15^N)FFARL(^13^C_6_,^15^N)FGGK-OH reconstituted into POPC:POPA:POPG:CL (65:15:15:5) liposomes, at a protein-to-lipid molar ratio 1:20. The symbol ‘-’ indicates unassigned chemical shifts.

| Compound | Atom/Region | δ^1^H (ppm) | δ^13^C (ppm) | Experiment |
| --- | --- | --- | --- | --- |
| Val149 | **α** | 4.068 | 61.973 | INEPT, HETCOR |
|  | **β** | 2.071 | 32.107 | INEPT |
|  | **γ1** | 0.875 | 20.531 | INEPT, HETCOR |
|  | **γ2** | - | - | - |
| Gly155 | **α** | 4.215 | 44.421 | HETCOR |
| Leu160 | **α** | 4.295 | 55.689 | INEPT |
|  |  | 3.853 | 56.482 | HETCOR |
|  | **β** | 1.081 | 41.615 | HETCOR |
|  | **δ1/2** | 0.549 | 23.927 | INEPT, HETCOR |
|  | **γ** | 1.137 | 25.907 | INEPT, HETCOR |
| POPC:POPA:POPG:CL | **ω** | 0.898 | 15.803 | INEPT |
|  | **ω-1** | 1.301 | **24.617** | **INEPT, HETCOR** |
|  | **ω-2** | 1.266 | 33.930 | **INEPT, HETCOR** |
|  | **Bulk** | 1.292 | 31.657 | **INEPT, HETCOR** |
|  | **C1** | - | - | - |
|  | **C2** | 2.324 | 36.034 | **INEPT, HETCOR** |
|  | **C3** | 1.570 | 26.942 | **INEPT, HETCOR** |
|  | **g1** | 4.328 | 64.968 | **INEPT, HETCOR** |
|  | **g2** | 5.259 | 72.584 | **INEPT, HETCOR** |
|  | **g3** | 3.987 | 65.792 | **INEPT, HETCOR** |
|  | **cis±1** | 2.018 | 29.235 | **INEPT, HETCOR** |
| POPC | α | 4.284 | 61.910 | **INEPT, HETCOR** |
|  | β | 3.687 | 68.085 | **INEPT, HETCOR** |
|  | γ | 3.237 | 56.076 | INEPT |
| POPG | α | - | - | - |
|  | β | 3.770 | 74.338 | INEPT |
|  | γ | 3.598 | 64.809 | INEPT |
| CL | α | - | - | - |
|  | β | 3.851 | 72.924 | INEPT |
|  | **dicis±1** | 2.742 | 27.370 | INEPT |
| Glycerol | **C1/C3** | 3.708 | 61.745 | INEPT |
|  | **C2** | - | - | - |

**Supporting Table 4**: MAS ssNMR parameter, Symbols: T_sample_ = sample temperature; ν_MAS_ = MAS frequency; τ_rd_ = recycle delay between scans; t_1,max_ = maximum t_1_ (indirect dimension 1) evolution time; t_1,inc_ = increment for t_1_ (indirect dimension 1) evolution time; d_mix_= PDSD mixing time; τ_dwell_ = dwell time during direct FID acquisition; τ_acq_ = maximum acquisition time during direct FID detection; ν_1H, acq_ = dipolar decoupling field strength during FID acquisition and indirect dimension evolution; τ_filter_ = initial t_2_ filter delay.

| 2D ^1^H-^13^C HETCOR | T_sample_ = 30°C; ν_MAS_ = 11 kHz, t_1,max_ = 5.5 ms; t_1,inc_ = 119 μs; τ_dwell_ = 9 μs; τ_rd_ = 2 s; τ_acq_ = 20 ms |
| --- | --- |

| 2D ^1^H-^13^C INEPT | T_sample_ = 30°C; ν_MAS_ = 11 kHz, t_1,max_ = 6 ms; t_1,inc_ = 138.8 μs; τ_dwell_ = 9 μs; τ_rd_ = 2 s; τ_acq_ = 18 ms |
| --- | --- |

| 2D ^13^C-^13^C PDSD | T_sample_ = 31°C/12°C /-1°C; ν_MAS_ = 11 kHz; d_mix_= 50 ms or 150 ms; t_1,max_ = 5 ms; t_1,inc_ = 48.7 μs; τ_dwell_ = 9 μs; τ_rd_ = 2 s; τ_acq_ = 20 ms |
| --- | --- |

| 2D ^13^C-^13^C DP-PDSD | T_sample_ = 30°C; ν_MAS_ = 11 kHz; d_mix_= 150 ms; t_1,max_ = 5 ms; t_1,inc_ = 48.7 μs; τ_dwell_ = 9 μs; τ_rd_ = 2 s; τ_acq_ = 20 ms |
| --- | --- |

| T_2_-filtered J-edited 2D ^1^H-^13^C HETCOR | T_sample_ = 30°C; ν_MAS_ = 11 kHz, τ_rd_ = 1.8 s, t_1,max_ = 11.0 ms; t_1,inc_ = 170.0 μs; τ_dwell_ = 9 μs; τ_acq_ = 20 ms; τ_SD_=100ms; τ_filter_=2.18 x 2 ms |
| --- | --- |

| 1D ^1^H-^13^C CP | T_sample_ = 31°C/20°C/15°C/-1°C; ν_MAS_ = 11 kHz; τ_rd_ = 2 s; τ_dwell_ = 9 μs; τ_acq_ = 20 ms |
| --- | --- |

| 1D ^1^H-^13^C INEPT | T_sample_ = 31°C/20°C/15°C/-1°C; ν_MAS_ = 11 kHz ; τ_rd_ = 2 s ; τ_dwell_ = 9 μs; τ_acq_ = 20 ms |
| --- | --- |

| **Supporting Table 5: Strains used in this study** | | | | | | | | | | | | |
| --- | --- | --- | --- | --- | --- | --- | --- | --- | --- | --- | --- | --- |
| Number | Strain | | Genotype | Source | | | Parent strain | | Construction (primers in Supplementary Table 6) | |  |  |
| NA | R800 | | R6 derivative | Gift from J.-P. Claverys (France) | | |  | |  | |  |  |
| Spn5 | WT | | R800 *rpsL1* | Gift from J.-P. Claverys (France) | | |  | |  | |  |  |
| Spn1556 | *ΔrocS::Janus* | | R800 *rpsL1,* *ΔrocS::kan-rpsL* | | | Mercy et al.[5] | |  | |  | |  |
| Spn1024 | *gfp-rocS* | | R800 *rpsL1, GFP-rocS* | Mercy et al.[5] | | |  | |  | |  |  |
| Spn1557 | *ΔrocS* | | R800 *rpsL1, ΔrocS* | Mercy et al.[5] | | |  | |  | |  |  |
| Spn4551 | *rocS_G155E_* | | R800 *rpsL1, rocS_G155E_* | This study | | | Spn1556 | | PCR 1113-1116 (PCR 1113-5484#Spn5 + PCR 5483-1116#Spn5) | |  |  |
| Spn4612 | *gfp-rocS_G155E_* | | R800 *rpsL1, gfp-rocS_G155E_* | This study | | | Spn1556 | | PCR 1113-1116 (PCR 1113-5484#Spn1024 + PCR 5483-1116#Spn1024) | |  |  |
| **Supporting Table 6: Primers used in this study** | | | | | | | | | | | | |
| Number | | Name | | | Sequence 5’-3' | | | | | | | |
| 1113 | | Fwd upstream of rocS | | | GTCTGCTATGAGTGTGGCGATTTTGGC | | | | | | | |
| 5484 | | RocS_G155E_Rv | | | CGAGCAAAAAATTCTTTTTTAGTGGATTGGAC | | | | | | | |
| 5483 | | RocS_G155E_Fw | | | GTCCAATCCACTAAAAAAGAATTTTTTGCTCG | | | | | | | |
| 1116 | | Rev downstream of rocS | | | CTACTTTCTGTCTCTAACAATTCCCTAG | | | | | | | |

**Supporting Table 7**: **Summary of experimental conditions for all figures**. This table summarizes the experimental parameters for each figure. Columns indicate: figure panel, technique, key parameters, sample (including peptide type, reconstitution into lipid membranes and peptide-to-lipid (P/L) ratio in mol/mol), buffer composition, temperature (°C) and additional notes. Peptide sequences: RocS MTS peptide selectively ^13^C: AKEEV(13C5,15N)QATKKG(13C2,15N)FFARL(13C6,15N)FGGK; RocS MTS: AKEEVQATKKGFFARLFGGK; and short RocS MTS: AKEEVQATKKGFFARLFG.

| Figure | Technique | Parameters | Sample | Buffer | Temperature | Note |
| --- | --- | --- | --- | --- | --- | --- |
| 2B | ssNMR | 2D ^13^C-^13^C PDSD  50 and 150 ms mixing times | RocS MTS peptide selectively ^13^C labeled reconstituted in  POPC:POPG:POPA:CL (65:15:15:5),  P/L = 1:20 | 25 mM Tris-HCl pH 7.5, 150 mM NaCl, 5% glycerol | 30 °C | See Supporting Table 4 |
| 2C |  | 2D ^1^H-^13^C HETCOR  and INEPT |  |  |  |  |
| 3A-B | ssNMR | T2-filtered J-edited 2D ^1^H-^13^C HETCOR | RocS MTS peptide selectively ^13^C labeled reconstituted in  POPC:POPG:POPA:CL (65:15:15:5),  P/L = 1:20 | 25 mM Tris-HCl pH 7.5, 150 mM NaCl, 5% glycerol | 31 °C | See Supporting Table 4 |
| 4A | ssNMR | ^2^H static ssNMR | RocS MTS/ short RocS MTS/negative control reconstituted in  POPC (doped with POPCd-31):POPG:POPA:CL (65:15:15:5), P/L = 1:20 | 25 mM Tris-HCl pH 7.5, 150 mM NaCl, 5% glycerol | 25 °C |  |
| 4B |  | ^31^P ssNMR |  |  |  |  |
| 4D | ssNMR | ^2^H static ssNMR | RocS MTS/ short RocS MTS/negative control reconstituted in  POPC (doped with POPCd-31),  P/L = 1:20 | 25 mM Tris-HCl pH 7.5, 150 mM NaCl, 5% glycerol | 25 °C |  |
| 4E |  | ^31^P ssNMR |  |  |  |  |
| 5A | ssNMR | 2D ^13^C-^13^C PDSD | RocS MTS peptide selectively ^13^C labeled, reconstituted in  POPC:POPG:POPA:CL (65:15:15:5),  P/L = 1:20 | 25 mM Tris-HCl pH 7.5, 150 mM NaCl, 5% glycerol | -1/12/31 °C | See Supporting Table 4 |
| 5B |  | 1D ^13^C CP |  |  | -1/15/20/31 °C |  |
| 5C |  | 1D ^13^C INEPT |  |  | -1/15/20/31 °C |  |
| 6A-G | AFM |  | Supported lipid bilayer of  POPC:POPG:POPA:CL (65:15:15:5) with 10 µM short RocS MTS | 25 mM Tris-HCl pH 7.5, 150 mM NaCl, 5% glycerol | 25 °C |  |

**Supporting Materials and Methods**

**Amino acid sequence alignment**

The amino acid sequences of RocS from *Streptococcus pneumoniae* was retrieved from the UniProt database (accession code: Q8DQ15). To facilitate comparative analysis, homologous RocS sequences from other *Streptococcus* species were identified and downloaded from the same database in FASTA format. Multiple sequence alignment was performed using Clustal Omega[6] (version 1.2.4on the EMBL-EBI web server with default parameters, including gap penalties and substitution matrix settings. The resulting alignments were visualized and analyzed using Jalview[7] (version 2.11.2.7). Sequence conservation and similarity were evaluated using the visualization and scoring tools provided within Jalview. MinD sequences from *Escherichia coli* strain K12 (P0AEZ3), *Salmonella enterica* subsp. enterica serovar Typhimurium str. LT2 (Q8ZP10), *Yersinia pestis* CO-92 (A0A5P8YEZ0), *Vibrio cholerae* M1526 (A0A5Q6PMH8), *Pseudomonas aeruginosa* PAO1 (Q9HYZ6), *Neisseria gonorrhoeae* CH811 (Q9AG19), *Xylella fastidiosa* strain M23 (B2I966), *Shewanella* sp (A0A9E6B3T8), *Listeria monocytogenes* CFSAN072502 (A0A823IWG3), *Clostridium ultunense* strain Esp (M1Z6E7), *Bacillus subtilis* 168 (Q01464), *Aquifex aeolicus* VF5 (O67033), *Methanocaldococcus jannaschii* strain ATCC43067 (Q57967), *Archaeoglobus fulgidus* SpSt-87 (A0A7C3RCL2), *Pyrococcus furiosus* strain ATCC 43587 (A0A5C0XTN6), *Borrelia maritima* CA690 (A0A5J6W9N7), *Treponema pallidum* Nichols (Q56340), *Arabidopsis thaliana* cv. Columbia (Q9MBA2) and *Oryza sativa* subsp. Japonica (Q0DF98) were retrieved from UniProt and aligned using T-Coffee[8] with default parameters. *Escherichia coli* MinD was used as the reference for conservation analysis. Residues identical to the reference were marked as conserved, and conservative substitutions were defined based on physicochemical similarity.

**References**

[1] Pott, T. and Dufourc, E.J. (1995). Action of melittin on the DPPC-cholesterol liquid-ordered phase: a solid state 2H-and 31P-NMR study. *Biophysical Journal*. https://doi.org/10.1016/S0006-3495(95)80272-9.

[2] Beck, J.G. *et al.* (2007). Plant sterols in “rafts”: a better way to regulate membrane thermal shocks. *The FASEB Journal*. https://doi.org/10.1096/fj.06-7809com.

[3] Dufourc, E.J. *et al.* (1992). Dynamics of phosphate head groups in biomembranes. Comprehensive analysis using phosphorus-31 nuclear magnetic resonance lineshape and relaxation time measurements. *Biophysical Journal*. https://doi.org/10.1016/S0006-3495(92)81814-3.

[4] Álvarez-Mena, A. *et al.* (2025). Bacterial flotillins as destabilizers of phospholipid membranes. *Biochimica et Biophysica Acta (BBA) - Biomembranes*. https://doi.org/10.1016/j.bbamem.2024.184399.

[5] Mercy, C. *et al.* (2019). RocS drives chromosome segregation and nucleoid protection in Streptococcus pneumoniae. *Nature Microbiology*. https://doi.org/10.1038/s41564-019-0472-z.

[6] Madeira, F. *et al.* (2024). The EMBL-EBI Job Dispatcher sequence analysis tools framework in 2024. *Nucleic Acids Research*. https://doi.org/10.1093/nar/gkae241.

[7] Waterhouse, A.M. *et al.* (2009). Jalview Version 2--a multiple sequence alignment editor and analysis workbench. *Bioinformatics*. https://doi.org/10.1093/bioinformatics/btp033.

[8] Notredame, C. *et al.* (2000). T-coffee: a novel method for fast and accurate multiple sequence alignment 1 1Edited by J. Thornton. *Journal of Molecular Biology*. https://doi.org/10.1006/jmbi.2000.4042.
